# Survivin promotes a glycolytic switch in CD4^+^ T cells by suppressing the transcription of PFKFB3 in rheumatoid arthritis

**DOI:** 10.1016/j.isci.2022.105526

**Published:** 2022-11-07

**Authors:** Malin C. Erlandsson, Karin M.E. Andersson, Nina Y. Oparina, Venkataragavan Chandrasekaran, Tibor Saghy, Anastasios Damdimopoulos, Maria-Jose Garcia-Bonete, Zakaria Einbeigi, Sofia T. Silfverswärd, Marcela Pekna, Gergely Katona, Maria I. Bokarewa

**Affiliations:** 1 Department of Rheumatology and Inflammation Research, Institute of Medicine, University of Gothenburg, Box 480, 40530 Gothenburg, Sweden; 2 Rheumatology Clinic, Sahlgrenska University Hospital, Gröna stråket 16, 41346 Gothenburg, Sweden; 3 Bioinformatics and Expression Analysis Core Facility, Department of Biosciences and Nutrition, Karolinska Institute, 141 41 Huddinge, Sweden; 4 Department of Medical Biochemistry and Cell Biology, Institute of Biomedicine, University of Gothenburg, 40530 Gothenburg, Sweden; 5 Department of Medicine and Oncology, Southern Älvsborg Hospital, 50182 Borås, Sweden; 6 Department of Oncology, Institute of Clinical Science at Sahlgrenska Academy, University of Gothenburg, 405 30 Gothenburg, Sweden; 7 Department of Clinical Neuroscience, Institute of Neuroscience and Physiology, University of Gothenburg, Box 440, 40530 Gothenburg, Sweden; 8 Department of Chemistry and Molecular Biology, Faculty of Science, University of Gothenburg, 405 30 Gothenburg, Sweden

**Keywords:** Biological sciences, Immunology, Transcriptomics

## Abstract

In this study, we explore the role of nuclear survivin in maintaining the effector phenotype of IFNγ-producing T cells acting through the transcriptional control of glucose utilization. High expression of survivin in CD4^+^T cells was associated with IFNγ-dependent phenotype and anaerobic glycolysis. Transcriptome of CD4^+^ cells and sequencing of survivin-bound chromatin showed that nuclear survivin had a genome-wide and motif-specific binding to regulatory regions of the genes controlling cell metabolism. Survivin coprecipitates with transcription factors IRF1 and SMAD3, which repressed the transcription of the metabolic check-point enzyme phosphofructokinase 2 gene *PFKFB3* and promoted anaerobic glycolysis. Combining transcriptome analyses of CD4^+^ cells and functional studies in glucose metabolism, we demonstrated that the inhibition of survivin reverted *PFKFB3* production, inhibited glucose uptake, and reduces interferon effects in CD4^+^ cells. These results present a survivin-dependent mechanism in coordinating the metabolic adaptation of CD4^+^T cells and propose an attractive strategy to counteract IFNγ-dependent inflammation in autoimmunity.

## Highlights

• Survivin-rich IFNγ producing CD4^+^T cells are dependent on anaerobic glycolysis
• Survivin binds regulatory chromatin accompanied by IRF1 and SMAD3
• Survivin complex represses the transcription of *PFKFB3* and promotes anaerobic glycolysis
• Inhibition of survivin activates *PFKFB3* and reduces IFNγ production in CD4^+^ T cells

## Graphical abstract

**Figure:**
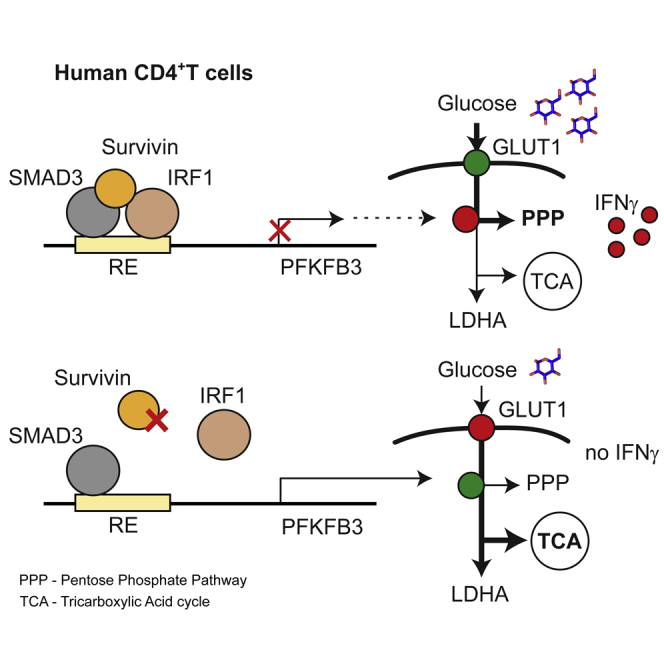

## Introduction

Activated CD4^+^ effector T cells are key players in autoimmune inflammation. These cells migrate, proliferate, and produce signal molecules at sites of inflammation to mobilize immunity. Production of IFNγ, the principal coordinator of adaptive immune responses in chronic inflammation, is the major characteristic feature of the effector T cells.^1^ To fuel effector responses, IFNγ producing cells undergo metabolic adaptation by switching glucose metabolism from entering the tricarboxylic acid (TCA) cycle to the pentose phosphate pathway of glycolysis thereby increasing availability of nucleotides, amino acids and fatty acids.^2^^,^^3^^,^^4^^,^^5^^,^^6^ The switch from TCA to a pentose phosphate-dependent utilization of glucose is an emergency act described in macrophages, T cells and neutrophils, which is maintained by through a high glucose consumption.^7^^,^^8^^,^^9^^,^^10^ IFNγ appeared to be particularly sensitive to cellular metabolic state and deletion of glucose transporter *GLUT1* and lactate dehydrogenese (*LDHA*) reduced IFNγ production.^11^^,^^12^ IFNγ dependent processes, such as inflammation and migration, initiated by the activation of IFNγ receptor, production of IFN-responsive factors (IRFs) and binding of the IRF-specific regulatory elements on chromatin to trigger the production of IFN-sensitive genes.^1^ Expression of the IFN-sensitive genes regulates the impact of IFNγ in pro-inflammatory effector functions, and in anti-TGFβ fibrotic processes that maintain autoimmunity.

Shared IFNγ-dependent processes are central for the pathogenesis of autoimmune diseases.^1^^,^^7^^,^^13^^,^^14^ The pleiotropy of IFNγ functions provides a broad spectrum of biological effects ascribed to this cytokine in different autoimmune diseases and even in different stages of the same condition alternating between immunostimulatory and immunosuppressive effects.^13^^,^^15^^,^^16^^,^^17^ Strategies to interfere with autoimmunity by targeting concordant changes in the expression of IFN-sensitive genes in blood leukocytes and target tissues may have broad therapeutic potential for immunological disorders. Inhibition of anabolic adaptation, which fuels IFNγ production, constitutes a promising approach toward mitigating the effects of IFNγ in autoimmunity.

Survivin, an oncoprotein encoded by *BIRC5,* is widely expressed in malignancies and during renewal of nonmalignant hematopoetic cells.^18^^,^^19^ Cytosolic and mitochondrial localization of survivin is tightly linked to its anti-apoptotic function,^20^ while nuclear localization of survivin has been attributed to its role in the chromosomal passenger complex^21^ and in the formation of macromolecular complexes, potentially supporting gene expression.^18^^,^^22^^,^^23^^,^^24^ Shuttling of survivin between cytosol and nucleus is assisted by exportin 1.^25^^,^^26^ Conditional deletion of survivin in hematopoietic progenitors^27^ and in thymocytes reduces mature CD4^+^ and CD8^+^ T cell populations^28^ and leads to a dysfunctional T-cell receptor and inability to mount a proper immune response to an antigen challenge.^29^ Survivin expression declines in mature T cells, but re-appears during critical phases of phenotype transition, such as the effector phenotype acquisition by CD4^+^ or CD8^+^ memory T cells.^30^ Accumulation of survivin in tissues and extracellular compartment is associated with severe autoimmune inflammation in rheumatoid arthritis,^31^ cutaneous psoriasis, and multiple sclerosis.^32^ Our earlier studies demonstrated that targeting survivin in experimental and clinical autoimmunity efficiently reduces inflammation, proliferation, and tissue damage.^33^^,^^34^^,^^35^^,^^36^^,^^37^ However, despite its importance in leukocyte development and disease, the role of survivin in the basic processes of T cell homeostasis has not been investigated.

In this study, we explored the role of nuclear survivin in maintaining the effector phenotype in IFNγ-producing Th1 cells acting through the transcriptional control of glucose utilization. To study this, we performed a genome-wide deep sequencing of survivin precipitated chromatin regions; identified survivin interactors on chromatin, and the biological processes regulated by survivin in cooperation with the identified interactors. Combining chromatin and transcriptome analyses with functional studies, we have searched for the genes sensitive to survivin inhibition and present a previously unknown survivin-dependent mechanism that coordinates metabolic adaptations during the activation of CD4^+^ T cells in autoimmunity.

## Results

### Survivin is an essential marker of the IFNγ-producing cell phenotype

**Figure 1:**
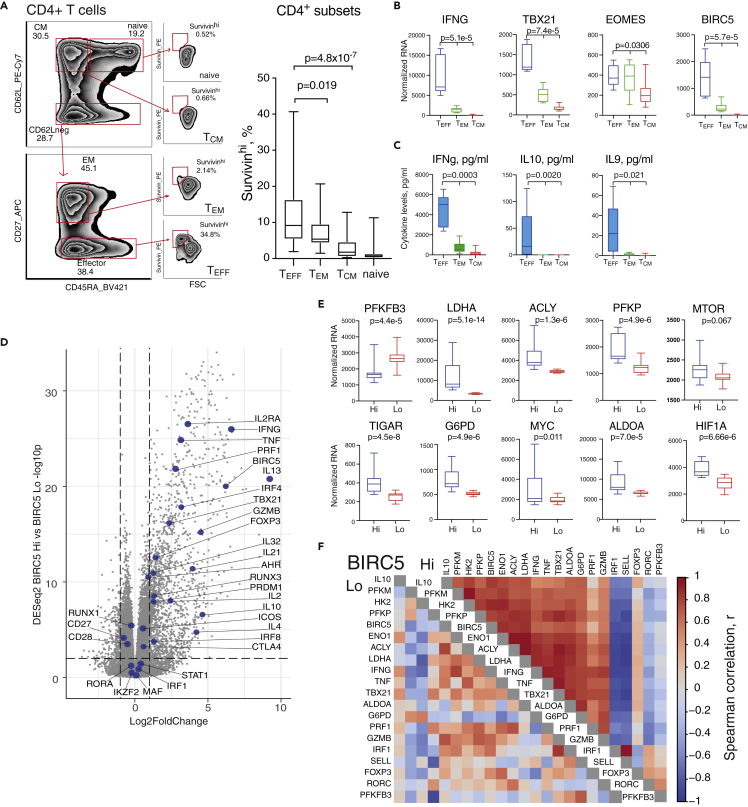
Survivin is essential for the phenotype of IFNγ-producing CD4^+^ T cells (A) Example of gating of naive (CD62L^hi^CD45RA^+^), central memory (CM, CD62L^hi^CD45RA^neg^), effector memory (EM, CD62L^neg^CD45RA^neg^CD27^hi^), and effector (EFF, CD62L^neg^CD45RA^+/−^CD27^neg^) populations of survivin^hi^CD4^+^ cells. Boxplot of survivin^hi^CD4^+^ cell frequency in 22 patients with RA. Boxes present IQR, line indicates median, and whiskers show min-to-max range. *p* values were determined by Wilcoxon test. (B) RNA-seq analysis was done on CD4^+^ cells of 24 patients with RA. Clustering of CD4^+^ RNA-Seq by the core genes characteristic of T-helper subsets resulted in T_EFF_, T_CM_, and T_EM_ clusters (Figure S1A). Boxplots of gene expression, by RNA-seq. Boxes present IQR, line indicates median, and whiskers show min-to-max range. *p* values were determined with DESeq2. (C) Boxplots of cytokines levels in supernatants, by ELISA.Boxes present IQR, line indicates median, and whiskers show min-to-max range. p values were determined by Kruskal-Wallis test (C). (D) Volcano plot of DE protein-coding genes in *BIRC5*^hi^ and *BIRC5*^lo^ cells analyzed by DESeq2. Th1 signature genes are indicated. (E) Boxplots of gene RNA-seq expression analysis of metabolic regulators and glycolytic enzymes in *BIRC5*^hi^ and *BIRC5*^lo^ (median split) of CD4^+^ cells sorted as above. Boxes present IQR, line indicates median, and whiskers show min-to-max range. p-values were determined with DESeq2. (F) Spearman correlation matrix of Th1 signature genes and the genes of glycolytic enzyme in *BIRC5*^hi^ and *BIRC5*^lo^ cells. Unsupervised clustering was done with corrplot Bioconductor in R-studio.

Survivin expressing CD4^+^T cells were identified by flow cytometry of the mononuclear leukocytes from the peripheral blood of 22 (16 female, 6 male) patients with rheumatoid arthritis (RA) (Table S1). The gating strategy of T cell subsets is shown in Figure 1A. We found that the effector cells (T_EFF_) defined as CD62L^neg^CD45RA^+/−^CD27^neg^ had higher levels of survivin than memory cells. On average, 9.2% (range 5.4–16.4%) of T_EFF_ cells contained survivin and had highest amount of survivin per cell (Figure 1A). A different set of CD4^+^ T cells isolated from 24 patients with RA (all female) was used to investigate the phenotype of survivin-producing CD4^+^T cells by RNA-seq analysis. Unsupervised clustering of the RNA-seq datasets by the core genes characteristic of T-helper subsets^38^ identified the accumulation of survivin/*BIRC5* in the T_EFF_ cluster marked by expression of Th1 signature genes (*e.g.*, *TBX21, EOMES, IL2RA,* and *IFNG*) (Figures 1B and S1A) and cytokines (IFNγ, IL9 and IL10) (Figure 1C), which correlated with *BIRC5* expression (Figure S1B). Comparison of *BIRC5*^hi^ and *BIRC5*^lo^ CD4^+^ cells revealed the complete Th1 signature to be enriched in the *BIRC5*^hi^ cells (Figure 1D).

Availability and efficient metabolism of glucose are required for IFNγ production and effector T cell function.^8^^,^^11^ Expression of the main glucose metabolism regulator HIF-1α differed between *BIRC5*^hi^ and *BIRC5*^lo^ CD4^+^ cells, but their expression of *MYC* and *MTOR* was similar (Figure 1E). Since *HIF1A* expression is controlled by hypoxia, the selective enrichment for *HIF1A* in *BIRC5*^hi^ cells prompted us to evaluate other genes of the hypoxia signature.^39^ We found that *BIRC5*^*hi*^ cells overexpress the canonical HIF-1α target genes, including lactate dehydrogenase (*LDHA*), enolase (*ENO1*), phosphoglycerate kinase 1 (*PGK1*), and aldolase A (*ALDOA*), associated with glucose metabolism (Figure S1C). Specifically, *BIRC5*^hi^ cells had a reduction in the key regulator of glucose processing the phospho-fructokinase 2, encoded by *PFKFB3* (Figure 1E) suggesting it’s deficiency. As a result, glucose was shunted to the pentose phosphate pathway, as reflected by increased expression of glucose-6-phosphate dehydrogenase (*G6PD*) and ATP citrate lyase (*ACLY*), favoring active fatty acid metabolism. The correlation matrix of the core Th1 genes and glycolysis markers revealed clear divergence in glucose utilization between *BIRC5*^hi^ and *BIRC5*^lo^ cells (Figure 1F). The tight interactions in *BIRC5*^hi^ cells suggested that survivin expression is functionally connected to these processes.

### Survivin-bound chromatin is annotated to regulators of glucose metabolism

**Figure 2:**
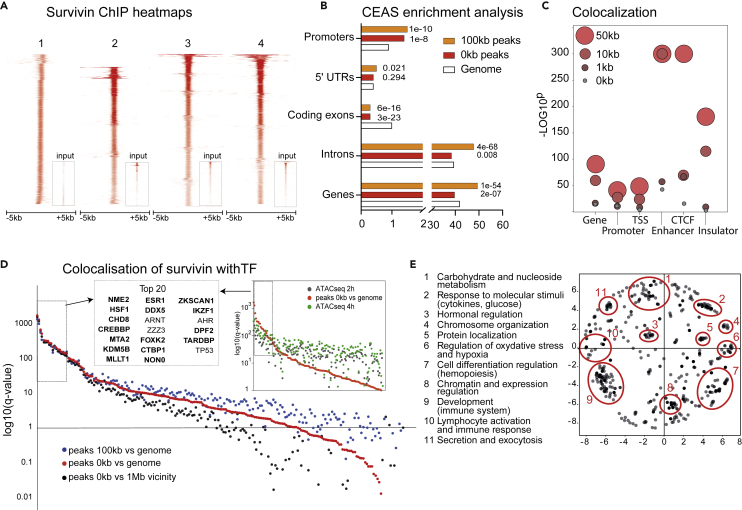
Binding of survivin to chromatin is predicted to regulate carbohydrate metabolism (A) Heatmap of survivin-ChIP-seq peaks from CD4^+^ T cells (n = 4, independent replicates originating from 12 individual CD4^+^ cell samples). (B) Bar plots of the distribution of survivin peaks (red bars, 0-kb flanks; orange bars, 100-kb flanks) compared to the genome (open bars). (C) Dot plot of enrichment significance for the colocalization of survivin peaks and DNA elements. *p* values were determined by two-tailed Fisher exact test. (D) Dot plot of individual q values for the colocalization of survivin and TF ChIP-seq peaks (ReMap2020). Red dots, 0-kb flanks, 10% overlap; blue dots, 100-kb flanks; black dots, 1-Mb genome neighborhood. Only TFs with >100 events are shown. **Inset** Dot plot of q values for the colocalization of TFs and survivin peaks in open chromatin regions. Common TFs are indicated in bold. (E) Semantic similarity map of Gene Ontology biological processes regulated by TFs that co-localized with survivin. Functional annotation was done in MetaScape. Dense GO:BP clusters are shown in ellipses and their functions are indicated. See complete list of GO:BP in Table S2.

Since survivin has been previously reported to bind to genomic DNA elements that regulate gene transcription,^22^^,^^23^^,^^24^ we performed the chromatin immunoprecipitation sequencing (ChIP-seq) analysis of 12 CD4^+^ cell cultures pooled in 4 replicates, which revealed 13704 nonredundant survivin-ChIP peaks (enrichment against input, adjusted p < 10^−5^) (Figure 2A). The peaks were unevenly distributed across the genome and were specifically accumulated in the chromatin areas within 10–100 kb distance from the *cis*-regulatory elements (RE) occupied by promoters, enhancers, chromatin insulator regions, and CTCF binding sites (Figures 2B and 2C).

To characterize the TF landscape of the survivin-ChIP peaks, we used the global ChIP-seq dataset for 1034 human transcriptional regulators in the ReMap database.^40^ We identified that binding sites of 146 TF candidates were significantly enriched across the survivin-ChIP peaks with 0 kb (minimal threshold for the overlapping peaks 10%) and 100-kb flanking regions as compared with the regions within 1 Mb around the peaks (Figure 2D). The q significance of association with survivin was higher for TFs in the regions of 0–100 kb and lower for TFs within 1Mb.

To identify TF binding sites in open chromatin of CD4^+^ cells, we used the ATAC-seq dataset (GSE138767^40^) to annotate unique nonredundant survivin-ChIP peaks. Analysis of the survivin-ChIP peaks within the open chormatin demostrates that survivin is tightly associated with a subset of TFs identified by the whole-genome analysis (Figure 2D**, inset**). The strength of the association defined by q-significance, did not differ between the chromatin regions accessible at 2 and 4 h. The top TFs identified by both analyses were those regulators of glucose and insulin metabolism, including CREBBP,^41^ KDM5B,^42^ FOXK2,^43^^,^^44^ CTBP1,^45^ and IKZF1.^46^

To identify biological processes regulated by the survivin-bound chromatin, we analyzed biological functions of the 146 TFs that co-localized with survivin peaks and annotated them to 2749 protein-coding genes expressed in CD4^+^ cells (RNA-seq, normalized raw counts >0.5) and to the Gene Ontology terms. This approach identified functional groups that regulate chromatin remodeling, protein modification, and metabolism (Figure 2E). Other functional groups regulated the response to hypoxia and organic substances, including glucose and cytokines (Table S2). These sets of analyses demonstrated that survivin was frequently located near the *cis*-REs and was functionally linked to the regulation of protein and carbohydrate metabolism.

### Survivin restricts *PFKFB3* expression and changes the metabolic requirements of CD4^+^ cells

**Figure 3:**
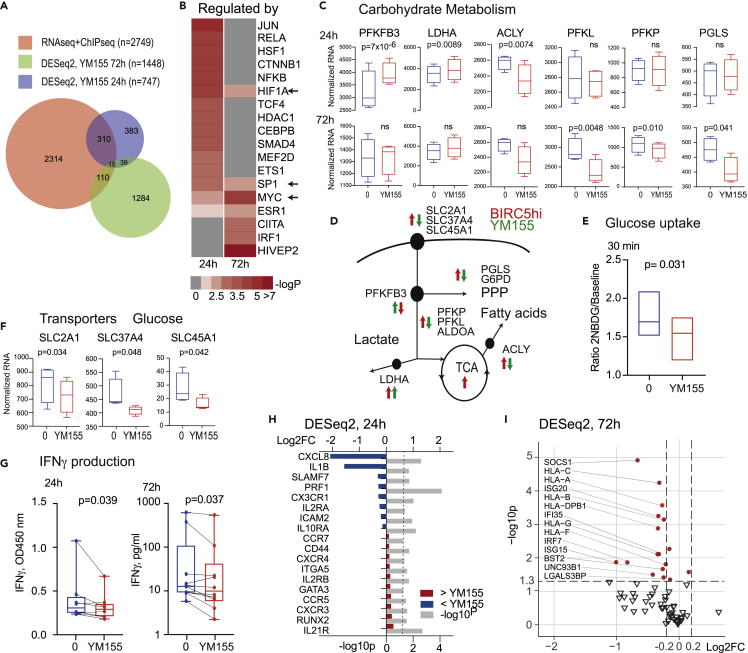
Survivin controls glycolysis through the phosphofructokinase metabolic axis RNA-seq of CD4^+^ T cells (n = 4) treated with anti-CD3 antibodies and the survivin inhibitor YM155 (0 and 10 nM) for 24 or 72 h and activated with IFNγ during the last 2 h. (A) Venn diagram of common protein-coding genes expressed in CD4^+^ cells (normalized RNA value > 1) and annotated to survivin peaks (red), and DE-Gs (nominal p < 0.05) in cells treated with YM155 for 24 (blue) or 72 h (green). (B) Heatmap of upstream trancriptional regulators of DE-Gs annotated with the TRUSST database. (C) Boxplots of gene expression by RNA-seq of glycolytic enzymes. Boxes present IQR, line indicates median, and whiskers show min-to-max range. p-values were obtained by DESeq2. (D) Schematic of glucose metabolism. Arrows indicate DE-Gs in BIRC5^hi^ cells (red) and YM155-treated cells (green). (E) Boxplots of 2NBD-glucose uptake by YM155-treated CD4^+^ cells (n = 4), normalized to baseline. Boxes prresent min-to-max range, line indicates median. p-values were determined by paired Wilcoxon’s sign-rank test. (F) Boxplots of the gene expression by RNA-seq. Boxes present IQR, line indicates median, and whiskers show min-to-max range. p-values were determined with DESeq2. (G) CD4^+^ T cells were activated with ConA/LPS in the presence of the survivin inhibitor YM155 (0 and 10 nM). Protein IFNγ levels were measured in supernatants after 24 h (n = 8) and 72 h (n = 10). Boxes present IQR, horizontal line indicates median, and whiskers show min-to-max range. Paired samples are connected with lines. p-values were obtained with Wilcoxon’s paired rank test. (H) Forest plot of the enrichment and p-values of IFN-sensitive DE-Gs at 24 h. (I) Volcano plot of IFN-sensitive DE-Gs at 72 h. Red dots indicate clinically relevant IFN-sensitive genes with p-value.

To investigate the role of survivin in the predicted biological processes, we used YM155 to inhibit survivin function^23^^,^^47^ in freshly isolated CD4^+^T cells. Cells were polarized with IFNγ for the final 2 h. Comparison of differentially expressed genes (DE-Gs) identified by RNA-seq analysis of YM155-treated (0 and 10 nM) CD4^+^ cells (nominal p < 0.05, DESeq2) with those annotated to survivin peaks showed that 11.8% (24 h) and 4.5% (72 h) of the protein-coding genes expressed in CD4^+^ cells were sensitive to survivin inhibition (Figure 3A). To identify TFs controlling the transcription of the DE-Gs, we used the curated TRRUST database. We found that the central metabolism regulators HIF-1α, c-MYC, and SP1 were among the transcriptional supervisors of the DE-Gs after 24 and 72 h of survivin inhibition. Other effects were attributed to the activity of SMAD4, JUN, NF-kB, RELA, ETS1 TFs at 24 h and to interferon regulatory factor 1 (IRF1) and the MHC class II transactivator at 72 h (Figure 3B).

To study in-depth engagement of survivin in the process of cellular glucose utilization, we analyzed YM155-treated and IFNγ-polarized CD4^+^ cells by RNA-seq. We found that mRNA levels of *PFKFB3* and *LDHA* increased rapidly in the YM155-treated cells (Figure 3C), indicating conversion of pyruvate into lactate, while *PGLS* and *ACLY* mRNA levels were decreased, indicating the downregulation of the pentose phosphate pathway and fatty acid metabolism (Figures 3C and 3D). These results show that survivin prevented the alterations in carbohydrate metabolism seen in the BIRC5^hi^ CD4^+^ cells from patients with RA (Figure 1F) but did not alter the mRNA levels of *HIF1A* or its metabolic targets *HK2*, *ALDOA*, *ENO1*, and *GAPDH* (Figure S2A).

To assess the role of survivin in glucose uptake by CD4^+^ cells, we measured the accumulation of fluorescently labeled D-glucose in CD4^+^ cell cultures (n = 4) activated with anti-CD3 antibodies combined with IFNγ. YM155 treatment reduced uptake of 2NBD-glucose (Figure 3E), presumably due to decreased expression of the HIF-1α-controlled sugar transporters GLUT1 (encoded by *SLC2A1*), glucose-6-phosphate translocase (*SLC37A4*), and proton-associated sugar transporter A (*SLC45A1*) (Figure 3F).

### Survivin inhibition resets TGFβ/SMAD signaling and promotes phenotype transition in CD4^+^T cells

As a consequence of the decreased glucose uptake and upregulation of PFKFB3 followed by the normalization of an aerobic glucose metabolism, we observed reduced IFNγ production by CD4^+^ cells treated with YM155 for 24 and 72 h (Figure 3G) and inhibition of IFN-dependent processes (Figure S2B). After 24 h, canonical IFN-sensitive genes were repressed, including cytotoxic *PRF1* and *GNL1*, the pro-inflammatory cytokines *CXCL8* and *IL1β*, and receptors that promote clonal T cell expansion (*IL2RA, SLAMF7*, *IL10RA)* and joint homing receptors (*CX3CR1, ITGB3, ICAM2, TREM25)* (Figure 3H). After 72 h of YM155 treatment, the downregulation of IFN-sensitive genes was even more pronounced, affecting multiple IRF1-dependent genes (*e.g.*, *SOCS1* and *HLA* family genes). Importantly, the IFN-sensitive genes included in autoimmunity signatures of RA,^48^ systemic lupus erythematosus^49^ and Sjögren’s syndrome^50^ (*e.g.*, *IRF7, GAS6, IFI35, IFITM2, ISG15, ISG20, ODF3B*) were also downregulated (Figure 3I).

**Figure 4:**
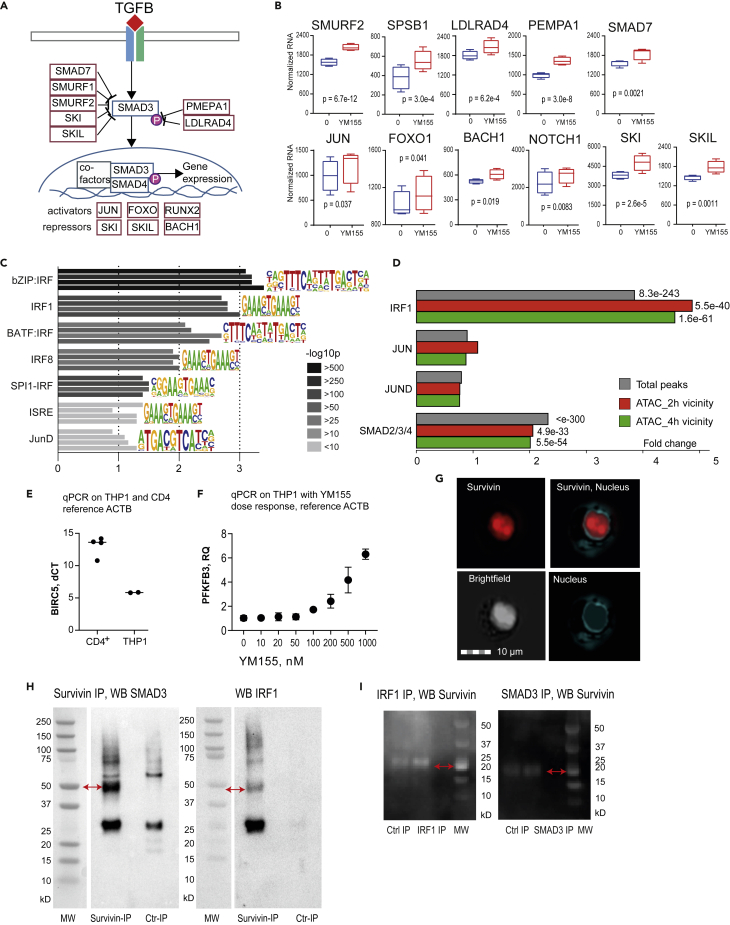
IRF1 and SMAD3 coordinate survivin binding to chromatin (A) TGFβ/SMAD signaling pathway. (B) Boxplots of TGFβ signaling mediators expression in RNA-seq of CD4^+^ T cells (n = 4) treated with anti-CD3 antibodies and the survivin inhibitor YM155 (0 and 10 nM) for 24 h and activated with IFNγ during the last 2 h. Boxes present IQR, line indicates median, and whiskers show min-to-max range. p-values were determined with paired DESeq2. (C) Bar plots of the enrichment and significance of IFNγ-relevant DNA motifs identified by *de novo* motif search in nonredundant survivin-ChIP peaks (enrichment against input, corrected p < 10^−5^) (n = 4 independent replicates), by JASPAR database. (D) Bar plots of motif enrichment in survivin peaks (0–10-kb flanks) in open chromatin regions. (E) BIRC5 expression in CD4^+^ and THP1 cells. Dots represent individual experiments. (F)PFKFB3 expression in THP1 cells treated with different concentrations of YM155. Dots represent individual experiments, error bars show standard deviation. (G)Survivin in the nucleus of THP1 cells depicted by imaging flow cytometry. Hoechst34580 staining was used for nucleus. Bright field shows cell morphology. (H) Western blots of THP1 nuclear extract before and after affinity immunoprecipitation with survivin, stained for IRF1, SMAD3. Rabbit IgG was used in non-targeting control IP. (I) Western blots of THP1 nuclear extract before and after immnoprecipitation with IRF1 and SMAD3, respectively, stained for survivin. Mouse IgG was used in non-targeting control IP. Arrows indicate bands corresponding to the protein of interest.

The TGFβ/SMAD pathway often counteracts the pro-inflammatory properties of IFNγ, and SMAD4 is a predicted upstream regulator of the DE-Gs (Figure 2B). We, therefore, investigated the effects of survivin inhibition on this pathway (Figures 4A and 4B) and found the upregulation of (1) the E3 ubiquitin ligases *SMURF2, SPSB1, SIAH3, LDLRAD4*, and *PMEPA1*, which facilitate proteolysis required for T cell reprogramming; (2) *SMAD7* and its co-repressors *SKI* and *SKIL*, which physically interact with the receptor-activated SMADs; and (3) the chromatin-binding SMAD3 co-factors *JUN, FOXO1,* and *BACH1* (Figure 4B). Notably, all those genes were among the most sensitive DE-Gs after survivin inhibition (nominal p < 0.005; Figure S3).

In agreement with the increased glycolytic activity of PFKFB3 and LDHA, which control the NOTCH1 and FOXO1 pathways,^51^^,^^52^^,^^53^ survivin inhibition increased mRNA levels of *FOXO1* and *NOTCH1* (Figure 4B). Consequently, CD4^+^ cells expressed higher levels of the surface receptors *CD44, IL21R, ITGA5,* and *CXCR3* downstream of NOTCH1 and the FOXO1 target genes *IL2RB, CCR5, CCR7*, and *CXCR4* (Figure 3H), which enabled the phenotype transition of CD4^+^ cells.

### Survivin colocalizes with IRF1 and SMAD3 on chromatin

After establishing survivin binding to chromatin, we sought to infer and validate associated protein partners through motif enrichment analysis in the region covered by survivin peaks. Using the JASPAR database of human TF, we discovered enrichment in IRF-binding motifs in all 4 independent ChIP-seq replicates. Predominant among the IRF motifs were IRF1 and IRF8, both containing the conserved GAAA repeat (Figure 4C). The survivin peaks were also enriched in the composite motifs AP1:IRF (AICE motif, GAAAnnnTGAc/gTCA) and SPI1:IRF (EICE motif, GGAAnnGAAA). Multiple binding sites for each motif were frequently present in a single survivin peak. The ISRE motif (GRAASTGAAAST), which bound two IRFs, was also enriched compared to the whole genome, yet infrequent within the survivin peaks (Figure 4C).

To connect survivin peaks with transcription, we annotated the whole set of unique survivin-ChIP peaks to open chromatin in human CD4^+^ cells activated with anti-CD3 and anti-CD28 antibodies, using ATAC-seq data (GSE138767^40^). We found that 12.3% (2 h) and 21.5% (4 h of cell stimulation) of the peaks were located within 0–10 kb of open chromatin regions (Figure S4A). An independent *de novo* motif search in those survivin peaks revealed up to 4.88-fold enrichment in the binding motifs of IRF1 and the SMAD3/SMAD4 complex, against the randomized background of all open chromatin (Figure 4C). No enrichment in JUND and JUN motifs was found. These findings confirmed the functional specificity of survivin binding.

To validate the colocalization of survivin with the anticipated TF partners identified by the bioinformatic analysis, we utilized the human monocytic cell line THP1 and found 50- to 150-fold higher spontaneous expression of survivin compared to the conA activated primary CD4^+^ cells (Figure 4E). Using immunocytochemistry with antibodies against survivin we found that survivin immunoreactivity almost exclusively in the nucleus (Figure 4G). Consistent with the findings made in primary CD4^+^T cells, inhibition of survivin with YM155 resulted in the upregulation of the *PFKFB*3 mRNA in THP1 cell culture (Figure 4F). We immunoprecipitated survivin from the total cell lysate and nuclear extract of THP1 cells using monoclonal rabbit-*anti*-human survivin antibodies and total rabbit IgG for control IP. Survivin-bound proteins were affinity isolated, heat denatured, and separated by electrophoresis. Western blotting of the nuclear extract showed that IRF1 and SMAD3 co-precipitated with survivin in three independent experiments (Figures 4H and S4D). Monoclonal antibodies to IRF1 identified a band of approximate size of 45 kDa corresponding to IRF1 with the calculated molecular weight of 37 kDa, which was not present in control IP. Antibodies targeting SMAD3 identified a band of approximate size of 50 kDa corresponding to SMAD3 with the calculated molecular weight of 48 kDa. Both, IRF1 and SMAD3 targeting antibodies revealed several additional bands, which varied in molecular weight and could be presumably explained by the presence of multiprotein complexes not resolved by disintegration step. No bands were identified in the material precipitated with control IgG (Figure 4H). Neither IRF8 nor c-MYC, JUN, or JUND (Figure S4D) was identified in the survivin IP material from those experiments.

To confirm reciprocally the observed co-precipitation of survivin with IRF1 and SMAD3 proteins, we performed an independent IP of THP1 nuclear material using antibodies to IRF1 and to SMAD3. Western blot of the IRF1-IP and SMAD3-IP with survivin antibodies revealed a band of approximately 20 kDa (Figure 4I), which corresponded to survivin protein monomer with molecular weight of 16.5 kDa.

Thus, survivin recruitment to open chromatin occurs through its interaction with IRF1 and SMAD3 in the regions containing sequence-specific motifs of those TFs (Figure 4D). These results provide molecular evidence that IRF1 and SMAD3 assist and coordinate the survivin-dependent transcriptional control which is described in the functional experiments.

### IRF1 and SMAD3 partner with survivin to regulate gene transcription

**Figure 5:**
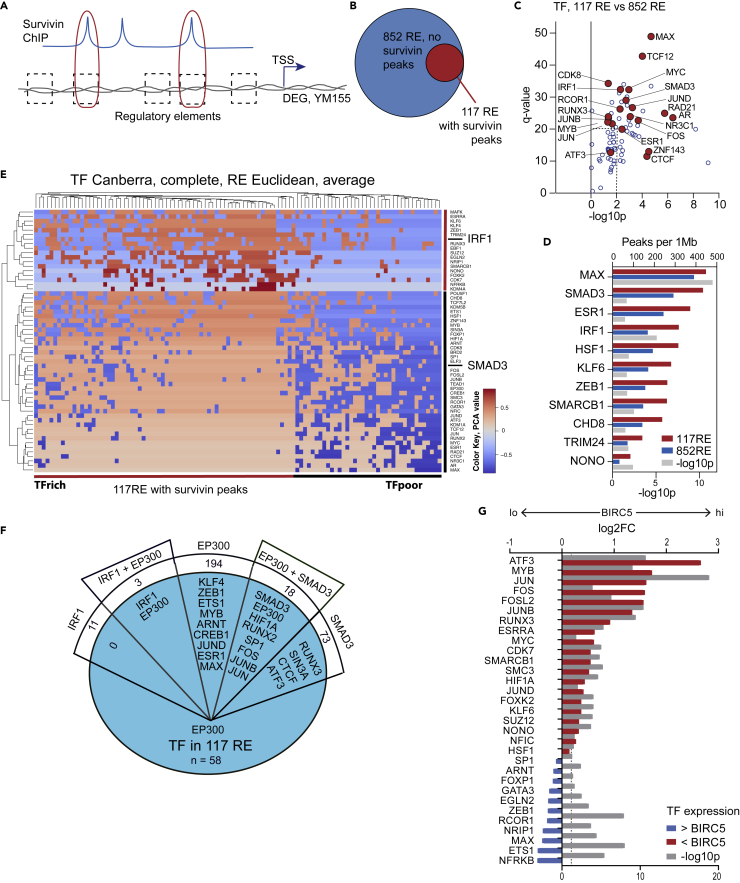
IRF1 and SMAD3 are predicted survivin partners in gene regulation (A) Selection of REs paired to protein-coding DE-Gs. (B) Venn diagram of all REs connected with DE-Gs (n = 969) and survivin-containing REs (0–10-kb flanks, n = 117). (C) Scatterplot of TFs enriched in survivin-containing REs against remaining REs (*x*-axis, -log10 p value) and the genome (*y* axis, q value). TFs in >75% of 117 REs are indicated. (D) Bar plot of TF density by ChIP-seq peaks (ReMap) in survivin-containing REs (red) and remaining REs (blue) and the significance (gray) of differences between them. (E) Heatmap of principal component analysis of enriched TFs in individual survivin-containing REs. REs were clustered by Euclidean distance and TFs by Canberra distance. Only TFs expressed in CD4^+^ cells (n = 58) were analyzed. (F) Protein-protein interaction by the BioGrid of IRF1, SMAD3, and EP300. (G) Forest plot of the enrichment and p values of TFs in BIRC5^hi^ versus BIRC5^lo^ CD4^+^ cells from 24 patients with RA. RNA-seq data were analyzed with DESeq2.

Since survivin-ChIP peaks accumulated in regulatory chromatin that was occupied by enhancers (Figure 2C), we analyzed the presence of survivin peaks in the *cis*-REs connected to the top protein-coding DE-Gs (Figure S3). Using the likelihood score for the enhancer-gene pairing,^54^ we identified 117 REs that were both connected to DE-Gs and associated with survivin peaks within 0–10 kb, and 852 REs with no survivin peaks (Figures 5A and 5B). These two groups of REs were similar in GeneHancer (GH) score, length/size of REs, and distance to the transcription start site (TSS) (Figure S5A).

Among the TF ChIP-seq peaks that co-localized with the survivin peaks (10% overlap, 0-kb flanks) in ChIP-seq datasets (Figures 2D), 58 TFs were expressed in CD4^+^ cells and were more abundant in survivin-containing REs compared to the whole genome and to the remaining REs (all p < 10^−3^) (Figure 5C). IRF1 and SMAD3 were among the most frequent and abundant survivin partners in REs connected to DE-Gs, as shown by density distribution analysis (Figure 5D). Principal component analysis of the enriched TF distribution across the REs, followed by unsupervised clustering of the components (Figure 5E) revealed that the REs clustered by the total density of TFs (TF-poor and TF-rich) rather than by gene association and further by the association of TFs around IRF1 or SMAD3 (Figure 5E). Thus, the immunoprecipitation of survivin with IRF1 and SMAD3 suggests its participation in TF complexes with distinct functions and diverse protein compositions. Using the BioGrid database to analyze protein-protein interactions, we identified histone acetyltransferase EP300 and glycogen synthase kinase 3B as the only common interactors of IRF1 and SMAD3 (Figure 5F). EP300, a protein that recruits TFs to distant enhancers, was enriched in survivin-containing REs and physically interacted with several other enriched TFs (Figures 5E and 5F), providing a broad platform for building multiprotein complexes. This prediction of multiprotein interactions also finds indirect support in the differential expression of the known IRF1 and SMAD3 interactors in *BIRC5*^hi^CD4^+^ cells of patients with RA (Figures 5G, S5B, and S5C).

### Survivin has a specific pattern of transcriptional regulation

**Figure 6:**
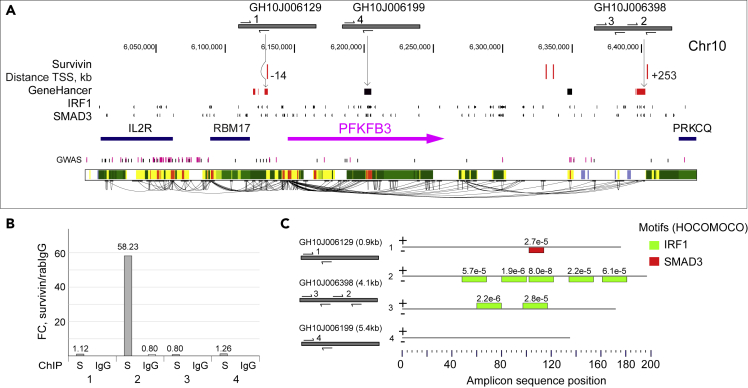
Chromatin-survivin interaction in the *PFKFB3* locus of CD4^+^ T cells (A) Genomic map of *PFKFB3*. Magenta indicates the position of the canonical gene transcript; arrow indicates transcription orientation. Red dashes at the top of each locus indicate the positions of survivin-ChIP peaks. Distances to TSSs are shown. Boxes indicate regulatory elements (REs) paired to the *PFKFB3* gene. Gray bars above the genomic site indicate REs by GeneHancer identifier and position of primers used to verify survivin binding in qPCR. Solid curved lines indicate integrated annotation of RE connections identified with GeneHancer. Red boxes indicate REs <10 kb from the survivin peak. Vertical lines indicate positions of ChIP-seq peaks for IRF1 and SMAD3 determined by ReMap2020. Vertical lines indicate positions of GWAS SNPs associated with metabolic and autoimmune triads according to NHGRI GWAS catalog. Functional chromatin segmentation for intact CD4^+^ cells (RoadMap ChromHMM. E043:CD4^+^CD25^−^) is shown at the bottom of each map. Green blocks indicate actively transcribed areas; yellow blocks indicate enhancers; red blocks indicate active promoters; white blocks indicate areas of repressed/poised chromatin. (B) Bar chart shows enrichment of the genomic regions in survivin IP compared to control IP with rabbit IgG, measured by qPCR and adjusted to input. (C) IRF1 and SMAD3 binding sites in the amplified genomic regions, identified using MAST tool from MEME Suite.

To explore the mode of survivin-specific transcriptional regulation, we analyzed chromatin regions containing genes highly sensitive to survivin inhibition. Several common features emerged, including (1) long-range interactions between survivin-containing REs and the promoters of target genes, (2) the location of survivin-containing REs among REs clustered into regulatory modules, and (3) the location of survivin-containing REs on repressed/poised chromatin. These features are clearly seen in three genes critical for survivin-dependent metabolism in CD4^+^ cells: *PFKFB3* (Figure 6A), *BIRC2,* and *SMURF2* (Figures S6A and S6B), all of which were transcriptionally activated by survivin inhibition.

Four survivin-ChIP peaks were associated with 5 high-scored REs paired to *PFKFB3* (Figure 6A). These REs covered a region extending from ∼20 kb upstream to 250 kb downstream of *PFKFB3*. According to the ReMap database, both the upstream and the downstream REs contained ChIP peaks for IRF1 and SMAD3 grouped together with the survivin peaks (Figure 5E). Three survivin-ChIP peaks were annotated to the REs connected to *BIRC2* and located ∼100 and ∼400 kb downstream of the TSS according to the GeneHancer database (Figure S6A). Despite their distant location, both REs were strongly linked to *BIRC2* (GH scores of 1.56 and 10.95, respectively) and according to the ReMap, contained multiple IRF1 and SMAD3 ChIP-seq peaks. Additionally, both RE were located within the repressed/poised chromatin according to the functional chromatin segmentation in CD4^+^ cells. Five survivin-ChIP peaks were found in the genomic region adjustent to the *SMURF2* gene (Figure S6B). Four of those peaks were annotated to the REs that formed a dense cluster spanning the region of ∼100 kb upstream of the TSS and built a higher-order regulatory unit at that site. Thus, the inhibition of survivin could trigger simultaneous activation of the clustered REs as predicted by the RoadMap data, which could explain the pronounced upregulation of *SMURF2* expression observed in the functional experiment (Figure 4B).

To further investigate survivin binding to the chromatin regions containing RE connected to *PFKFB3*, we performed a ChIP experiment targeting survivin in THP1 cells. To amplify the survivin-bound chromatin fraction in the genomic loci that overlap REs and survivin peaks, we designed a set of specific primers within the enhancers connected to the *PFKFB3* gene (Figure 6A. Table S3A). Using conventional qPCR, survivin ChIP of THP1 cells was amplified in four independent regions within REs GH10J006129, GH10J006398, and GH10J006199. After adjustment to the PCR results in ChIP with total rabbit IgG, we found that survivin IP was significantly enriched within the central part of RE GH10J006398 (site 2), while the regions in REs GH10J006129 and GH10J006199 had no such enrichment (Figure 6B). The TF motif analysis of the amplified regions in RE GH10J006398 identified multiple binding sites of IRF1, and accumulation of SMAD3 binding motifs in RE GH10J006129 (Figure 6C). In contrast, the amplified region in RE GH10J006199 contained no binding motifs of these TFs, supporting the observations from bioinformatic and physical interaction experiments. These findings confirm the specificity of survivin binding to the chromatin of THP1 cells and reproduced the results obtained in the survivin ChIP-Seq experiments on CD4^+^ T cells. Thus, survivin binding to these REs may control glucose utilization in T cells through the regulation of *PFKFB3* expression.

## Discussion

This study demonstrates a survivin-dependent mechanism of metabolic adaptation existing in the IFNγ-producing CD4^+^ cells. We show that nuclear survivin exhibits genome-wide and motif-specific binding to chromatin with unappreciated function in gene transcription control. The exact position of survivin binding is defined here by its physical interaction with the sequences of *cis*-RE and the TFs IRF1 and SMAD3/SMAD4. We show that survivin accompanied by IRF1 and SMAD3 keeps control of *PFKFB3*, the major point of metabolic adaptation for autoreactive T cells, and other genes responsible for glycolysis and sugar transport. Thus, survivin binding to chromatin acts as an epigenetic check-point coordinating a metabolic switch required for the effector function of the IFNγ-producing CD4^+^ cells.

This study demonstrated a solid reciprocal connection between survivin and IFNγ expression in the clinical material of patients with RA and in healthy CD4^+^T cell cultures. Previous studies reported that the activation of IFNγ signaling induced survivin transcription through STAT1 binding to the survivin/*BIRC5* gene promoter.^55^ Stimulation of human T cells with survivin peptides induced IFNγ production.^56^ Concordant with our results, inhibition of survivin with YM155 in human T cells led to a significant reduction in IFNγ production.^57^ We did not find any evidence for transcriptional control of the *IFNG* gene by survivin. Instead, survivin mediates IFNγ effects and regulates metabolic genes acting as an IRF1 partner.

We show that the repression of the key glycolytic enzyme PFKFB3 is central to the survivin-dependent metabolic effects in CD4^+^ cells. It leads to the activation of LDHA and aerobic glycolysis and a cessation of the pentose phosphate pathway. Expression of *PFKFB3* is altered in response to growth factors, inflammation, and ischemia, all of which activate estrogen receptor-, hypoxia-, or progesterone response elements on its promoter.^58^ Maintenance of *PFKFB3* repression requires energy. Integrative analysis of ChIP-seq and protein binding data identified IRF1/survivin/SMAD3 complex as a potent repressor of the REs connected to *PFKFB3*. Inhibition of survivin increased *PFKFB3* expression and restored conventional aerobic glycolysis through the TCA cycle, which reduced the glucose uptake and IFNγ production. This survivin-dependent change in the mode of glucose utilization is consistent with the logical connection between survivin, and IRF1-dependent effector function of CD4^+^ cells. Survivin is frequently bound to chromatin sequences containing IRF motifs and directly binds IRF1, the lineage-specific TF that mediates IFNγ signaling, enabling the transcriptional control of IRF1 target genes. Inhibition of survivin significantly impaired both IFNγ production and the sensitivity of CD4^+^ cells to IFNγ stimulation, which is required to maintain their effector phenotype and chronic inflammation.

Our findings showed that survivin represses TGFβ/SMAD-dependent processes in CD4^+^ cells. Indeed, genes downstream of TGFβ/SMAD were among the top DE-Gs upregulated after survivin inhibition, and SMAD3 was one of the most densely present TFs in the REs of those DE-Gs. Finally, our findings in the immunoprecipitation studies revealed a close interaction between survivin and SMAD3/4. JUN did not interact with survivin in western blot and was not enriched in the survivin peaks in open chromatin; but neither of those findings excludes the possibility of an interaction between AP-1 TFs and SMAD3^59^ or their consolidating effect on the survivin/SMAD3 binding complex. Likewise, SMAD3/4 and AP1 proteins are frequently found on distant *cis*-REs, where they facilitate promoter-enhancer interactions through chromatin looping and triggering transcription.^60^ Cell activation with TGFβ elicits a widespread SMAD-dependent increase in chromatin accessibility.^61^ Hypothetically, formation of the survivin/SMAD3 complex might anchor SMAD3 to inactive/poised chromatin, creating a predisposition for rapid changes in transcriptional activity, as observed in our study. EP300 and CREB1 were the only common interactors for IRF1 and SMAD3. The binding sites for both TFs were significantly enriched in REs connected to the DE-Gs upregulated after survivin inhibition. In this scenario, survivin acts as a guardian of the functional chromatin state by preventing the EP300/CREB1 complex interaction with SMAD3. Remarkably, the activity of EP300/CREB1 is mediated by glucose^62^ and integrates the immune processes initiated by IFNγ^63^^,^^64^ and TGFβ-signaling, potentially by patronizing the transcriptional activity of the IRF1/survivin and survivin/SMAD3 complexes.

In agreement with our findings, repression of *PFKFB3,* which switched glucose processing to the pentose phosphate pathway, has been suggested as the major point of metabolic adaptation for T cells in RA contributing to autoimmunity^65^ and the invasive phenotype of synovial fibroblasts.^66^ In contrast to RA, autoimmune conditions such as type 1 diabetes, multiple sclerosis, and systemic lupus erythematosus utilize the pyruvate kinase-dependent hyperproduction of lactate to meet their energy demands and could experimentally be improved with the inhibition of PFKFB3.^67^^,^^68^^,^^69^ Analyses of publicly available datasets (*e.g.* HiC, eQTL) indicated a strong internal connection between the REs with survivin ChIP peaks and the *PFKFB3* promoter region. Multiple critical SNPs associated with autoimmune diabetes, RA, and celiac disease were discovered by GWAS in the *PFKFB3* genomic region close to the survivin binding sites. This strongly linked the region to metabolic and autoimmune conditions through variation in T cell transcription^70^^,^^71^^,^^72^ and lends relevance to the transcriptional control in the *PFKFB3* gene region for the development of autoimmune conditions.

In summary, our study identifies a previously unknown epigenetic mechanism that connects oncoprotein survivin with the effector phenotype of IFNγ-producing CD4^+^T cells. This occurs through the regulation of glucose utilization and transcriptional control of the *PFKFB3* locus. The tight interaction with the IRF1/survivin or survivin/SMAD3 complexes maintains expression of IFN-sensitive genes that are clinically relevant in several autoimmune diseases, including RA,^48^^,^^64^ systemic lupus erythematosus,^49^ and Sjögren’s syndrome.^50^ Our findings provide an insight in the fundamental role of survivin in bridging the transcriptional programs governed by IRF1 and SMAD3 in the regulation of the balance between IFNγ- and TGFβ-dependent processes. This knowledge could have direct practical application for patients. Mapping of metabolic state in CD4^+^ T cells could be used to personalize treatment choice and reduce drug resistance. Pharmacological interventions that selectively target the molecular interactions of survivin could be an attractive approach to improve control of IFNγ-dependent autoimmunity and treatment of RA.

### Limitations of the study

Our study has some limitations. We show that high levels of survivin in CD4^+^ cells result in low expression levels of PFKFB3 in the IFNγ producing cells. The connection between the expression, protein, and functional levels of the phospho-fructokinase 2, coded by PFKFB3 needs more detailed studies. Immunoprecipitation experiments demonstrate the presence of survivin, IRF1, and SMAD3 in the pulled-down material. This finding calls for structural studies to confirm the direct interaction between those proteins in a complex and to deduct the nature of this interaction. Finally, the study does not address the specificity of the YM155 inhibitory effect. Sepantronium bromide (YM155), a small-molecule that specifically suppresses survivin expression but not the expression of cIAP2, XIAP, Bcl-2, Bcl-XL, Bad,^47^ cIAP1, p53 or Stat3.^73^ Later studies indicated side effects and secondary targets of YM155 ^74-76^. In the light of our findings, unexpected translocation of TF SP1, ILF/NF110, and NF-κB heterodimers during YM155 treatment^74^^,^^75^ could be explained by previously unappreciated survivin binding to chromatin as a part of large TF complexes described by our studies. Concordant with Sim et al.,^76^ we observed a significant upregulation of FOXO1 and CYLD in YM155 treated CD4^+^ cells, which could be explained by survivin binding to RE connected to these genes. Thus, the results of this study support survivin-targeting specificity of YM155, but this assumption needs further investigations.

## STAR★Methods

### Key resources table

**Table:** 

REAGENT or RESOURCE	SOURCE	IDENTIFIER
**Antibodies**		

**Antibodies targeting survivin**		
Flow cytometry	RnD Systems	Polyclonal rabbit IgG 91630, PE; RRID: AB_2064066
ChIPseq, CD4	Santa Cruz Biotechology	Polyclonal rabbit IgG 10811; RRID: AB_2227956
ChIP, THP1	RnD systems	Polyclonal rabbit IgG AF886; RRID: AB_355684
IP, THP1	Abcam	Monoclonal rabbit IgG ab192675; RRID: AB_2064068
Western blot, THP1	Capra, Halmstad, Sweden	Polyclonal goat
**Antibodies in conventional and imaging Flow Cytometry**		
CD4	BD	SK3, APCH7; RRID: AB_1645732
CD8	BD	SK1, PerCP; RRID: AB_400280
CD62L	BD	DREG56, PECy7; RRID: AB_395929
CD27	BD	L128, APC; RRID: AB_647368
CD19	BD	HIB19, V500; RRID: AB_10562391
CD45RA	BioLegend	HI100, BV421; RRID: AB_10900421
Isotype control	RnD Systems	mouse IgG1κ, PE; RRID: AB_357344
Human Fc-block	BD	Fc1; RRID: AB_2728082
**Antibody used for cell stimulation**		
Anti-CD3	Sigma-Aldrich	OKT3; RRID: AB_2619696
**Antibodies used in immunoprecipitations and Western blots**		
Isotype control IP, total rabbit IgG	Abcam	ab171870; RRID: AB_2687657
Isotype control IP, mouse IgG1	BioLegend	400102; RRID: AB_2891079
Secondary antibody rabbit-anti-goat-HRP	Invitrogen	611620; RRID: AB_2533922
mouse-anti-human IRF1	Santa Cruz Biotechnology	H-8, sc-74530; RRID: AB_2126826
mouse-anti-human IRF8/ICSBP	Santa Cruz Biotechnology	E-9, sc-365042; RRID: AB_10850401
mouse-anti-human JUND	Santa Cruz Biotechnology	D-9, sc-271938; RRID: AB_10650101
mouse-anti-human SMAD3	Santa Cruz Biotechnology	38-Q, sc-101154; RRID: AB_1129525
mouse-anti-human MAX	Santa Cruz Biotechnology	H-2, sc-8011; RRID: AB_627913
mouse-anti-human MYC	Santa Cruz Biotechnology	9E10, sc-40; RRID: AB_627268
Secondary antibody sheep-anti-mouse-HRP	GE Healthcare	NA931; RRID: AB_772210

**Biological samples**		

Human CD4^+^ T cells, RA patients	This paper	N/A
Human CD4^+^ T cells, healthy controls	This paper	N/A
THP1	ATCC, Manassas, VA, USA	TIB-202

**Chemicals, peptides, and recombinant proteins**		

YM155, serpantronium bromide	Selleck Chemicals	S1130
Lymphoprep	Axis-Shield PoC As	LYS3773
RPMI	Gibco	21870-070
β-mercaptoethanol	Gibco	31350-010
Glutamax	Gibco	A12860-01
Gentamicin	Sanofi-Aventis	stock, 40 mg/mL
Fetal bovine serum	Sigma-Aldrich	F7524
Recombinant IFNγ	Peprotech	SKU: 300-02
Concanavalin A (ConA)	MP biomedicals	11492082
Lipopolysaccharide (LPS)	Sigma-Aldrich	L2880. *E.coli*, O111B4
Hoechst 34580	Molecular probes	H21486
PAGE	Novex	NuPage 4–12% Bis–Tris gels
Diflouride membranes	Invitrogen	iBlot
Development, WB	Amersham	ECL Select Western Blotting Detection Reagent
3,3,5,5-Tetramethylbenzidine, TMB	Sigma Aldrich	1 mg tablets

**Critical commercial assays**		

Positive selection of human CD4	Invitrogen	11331D
Cytofix-Cytoperm permeabilisation	BD	554722
Chromatin isolation	Qiagen	EpiTect ChIP OneDay
mRNA extraction	Norgen	Total micro mRNA kit
cDNA kit	Applied Biosystems	#4368814
SyBR green qPCR mastermix	Qiagen	330522
Lysis buffer for immunoprecipitation	Pierce	87787
Protease inhibitors	Roche	Complete Mini
Dynabeads protein G IP beads	Invitrogen	10007D
IFNγ ELISA	Sanquin	PelikineM1933
IL10 ELISA	RnD Systems	DY217B
IL9 ELISA	RnD Systems	DY209
Glucose uptake assay	Abcam	2NBDG kit

**Deposited data**		

Survivin ChIP-seq, human CD4^+^ T cells	This paper	GEO: GSE190354
RNA-seq, human CD4^+^ T cells	This paper	GEO: GSE190349
RNAseq, human CD4^+^ T cells YM155-treated, 24h	This paper	GEO: GSE190352
RNAseq, human CD4^+^ T cells YM155-treated, 72h	This paper	GEO: GSE190351
ATAC -seq, human CD4^+^ cells	Yang et al., 2020	GEO: GSE138767

**Oligonucleotides**		

qPCR primer	Sigma-Aldrich	Tables S3A and S3B

**Software and algorithms**		

DESeq2 (v.1.4.0)	Bioconductor	https://bioconductor.org/packages/release/bioc/html/DESeq2.html
EnhancedVolcano (v.1.4.0	Bioconductor	https://bioconductor.org/packages/release/bioc/html/EnhancedVolcano.html
Corrplot (V.0.85)	CRAN	https://cran.r-project.org/web/packages/corrplot/index.html
Factoextra	CRAN	https://cran.r-project.org/web/packages/factoextra/index.html
STARaligner	Dobin, et al. 2013, Github	https://github.com/alexdobin/STAR
HOMER	Heinz, et al. 2010,	http://homer.ucsd.edu/homer/
JASPAR	Khan, et al. 2018	https://jaspar.genereg.net/
ENSEMBL regulatory build	v103, 2020	http://www.ensembl.org/info/docs/funcgen/regulatory_build.html
ENCODE	v5, 2020	https://screen.encodeproject.org/https://api.wenglab.org/screen_v13/fdownloads/GRCh38-ccREs.CTCF-only.bed file
GeneHancer	v4.4	https://www.genecards.org/GeneHancer_version_4-4
Galaxy Suit		https://usegalaxy.org/
Cistrome Galaxy	CEAS v0.9.8; accessed November 1, 2020	http://cistrome.org/ap/root
Bedtools Suite	Github, accessed accessed 01feb2021–15apr 2021	https://github.com/arq5x/bedtools2
ReMap database	accessed November 15, 2020	http://remap.univ-amu.fr/
ReMap enrich	Github accessed November 15, 2020	https://github.com/remap-cisreg/ReMapEnrich
Gene Ontology Biological Processes	GO:BP	
ReViGo	accessed Dec 1, 2020	http://revigo.irb.hr/
GWAS		http://genome.ucsc.edu/).
NIH Roadmap Epigenomic Project	accessed March 22, 2022	http://epigenomegateway.wustl.edu/browser/roadmap/
GEWAS		http://genome.ucsc.edu/).

### Resource availability

#### Lead contact

Further information and requests for resources and reagents should be directed to and will be fulfilled by the lead contact, Maria Bokarewa (maria.bokarewa@rheuma.gu.se).

#### Materials availability

This study did not generate new unique reagents.

### Experimental model and subject details

Blood samples of 46 RA patients (22 + 24) and 7 healthy female controls were collected at the Rheumatology Clinic, Sahlgrenska Hospital, Gothenburg. Clinical characteristics of the patients are shown in Table S1. All RA patients fulfilled the EULAR/ACR classification criteria^77^ and gave written informed consent before the blood sampling. The study was approved by the Swedish Ethical Review Authority (659-2011) and done in accordance with the Declaration of Helsinki. The trial is registered at ClinicalTrials.gov (ID NCT03449589).

In this study, the peripheral blood mononuclear cells (PBMC) of RA patients were used for the flow cytometry. CD4^+^ cells isolated from PBMC were used for RNAseq, and ChIPseq.

CD4^+^ cells of healthy controls were used for ChIP-seq, RNA-seq after YM155 treatment, glucose uptake assay, and IFNγ production.

### Method details

#### Isolation and stimulation of CD4^+^ cells

Human peripheral blood mononuclear cells were isolated from venous peripheral blood by density gradient separation on Lymphoprep (Axis-Shield PoC As, Dundee, Scotland). CD4^+^ cells were isolated by positive selection (Invitrogen, 11331D), and cultured (1.25 × 10^6^ cells/mL) in wells coated with anti-CD3 antibody (0.5 mg/mL; OKT3, Sigma-Aldrich, Saint Luis, Missouri, USA), in RPMI medium supplemented with 50μM b2-mercaptoethanol (Gibco, Waltham, Massachusetts, USA), Glutamax 2 mM (Gibco), Gentamicin 50 μg/mL (Sanofi-Aventis, Paris, France) and 10% fetal bovine serum (Sigma-Aldrich) at 37°C in a humidified 5% CO_2_ atmosphere. Cells were treated with survivin inhibitors serpantronium bromide, YM155^47^ (YM155, Selleck Chemicals, Houston, TX), as indicated. The cells were stimulated with recombinant IFNγ (50 ng/mLl; Peprotech, Cranbury, NJ, USA) during the last 2 h and harversted for RNA-seq. Supernatants were used to measure cytokine levels.

#### Flow cytometry

Freshly isolated PBMC were stained for flow cytometry as described^22^ using antibodies to the following human surface antigens. Cells were then fixed and permeabilized with a Cytofix-Cytoperm fixation/permeabilization kit (BD) and stained with anti-survivin (91630, R&D Systems, Minneapolis, MN, USA) and isotype control (mouse IgG1κ, R&D Systems). The cells were collected in FACSCantoII flow cytometer (BD), and the data were analyzed with FlowJo software (BD, v.10.7) and fluorescence minus one controls.

#### Imaging flow cytometry

Fresh THP1 cells were pelleted by centrifugation and permeabilized with Cytofix-Cytoperm (BD). Non-specific binding was blocked using Fc-block reagent (BD 464220) and cells were stained with PE-conjugated anti-survivin antibodies (91630, R&D System) for 30 min at room temperature. Cells were washed and nuclei were stained with DNA dye Hoechst 34580 (5 ng/mL). The stained cells were collected into the imaging flow cytometer (ImageStreamX, MKII, Amnis) using 40× objective. Approximately 20,000 single-cell events were acquired and analyzed using IDEAS v.6.2 software (Amnis). Survivin nuclear translocation was analyzed using the nuclear translocation wizard in IDEAS™ (v6.2) analysis software. This wizard calculates the mean similarity of a nuclear probe (Hoechst) and a translocating probe (Survivin) using Pearson’s Correlation Coefficient. A mean similarity greater than or equal to 1 (R1 ≥ 1) indicates nuclear localization. The feature used in IDEAS™ to determine similarity was Similarity_Dilate(Object(M01, Ch01, Tight),1)_Ch03-Ch01. This included the morphology mask Dilate(Object(M01, Ch01, Tight),1) for a channel as well as the (M03, Ch03, Tight) mask for Survivin, both of which are generated by the nuclear translocation wizard in the IDEAS™ software (v6.2) and are based on previously published work.^78^

#### Chromatin immunoprecipitation (ChIPseq)

For ChIP-seq analysis, CD4^+^ cells isolated from 12 women were stimulated with concanavalin A (ConA, 0.625 μg/mL, Sigma-Aldrich), and lipopolysaccharide (LPS) (5 μg/mL, Sigma-Aldrich) for 72 h and pooled in 4 independent samples for DNA purification. The cells were cross-linked and lysed with the EpiTect ChIP OneDay kit (Qiagen), as recommended by the manufacturer. After sonication to shear the chromatin, cellular debris was removed by pelleting. After pre-clearing, 1% of the sample was saved as an input fraction and used as background for nonspecific chromatin binding. Pre-cleared chromatin was incubated with 2 μg of anti-survivin (10811, Santa Cruz Biotechnology, Santa Cruz, CA, USA). The immune complexes were washed, the cross-links were reversed, and the DNA was purified with the EpiTect ChIP OneDay kit (Qiagen) as recommended by the manufacturer. The quality of purified DNA was assessed with TapeStation (Agilent, Santa Clara, CA, USA). DNA libraries were prepared with ThruPLEX (Rubicon) and sequenced with a Hiseq2000 sequencing system (Illumina) according to the manufacturer’s protocols. Bcl-files were converted and demultiplexed to fastq with bcl2fastq (Illumina).

#### Transcriptional sequencing (RNA-seq)

RNA from CD4^+^ cells stimulated with anti-CD3 antibodies (0.5 mg/mL) and IFNg (50 ng/mL) was prepared with the Norgen Total Micro mRNA kit (Norgen, Ontario, Canada). Quality control was done with a Bioanalyzer RNA6000 Pico on an Agilent2100 (Agilent, St.Clara, CA, USA). Deep sequencing was done by RNA-seq (Hiseq2000, Illumina) at the LifeScience Laboratory, Huddinge, Sweden. Raw sequence data were obtained in Bcl files and converted to fastq text format with bcl2fastq. RNA-seq results were validated by qRT-PCR as described below.

#### Conventional qPCR

To validate results of survivin ChIP-seq, primers were designed to cover the peak region in the REs connected to the *PFKFB3* gene (Figure 6A). The RE with no survivin peak was used as a negative control. Survivin-ChIP material of THP1 cells was prepared as described below and analyzed in qPCR using primers presented in Table S3A. Amplification was calculated against the input by the ddCt method and thereafter adjusted to control IP using rabbit IgG (Dako).

RNA was isolated with the Total RNA Purification Kit (17200, Norgen Biotek). RNA concentration and quality were evaluated with a NanoDrop spectrophotometer (Thermo Fisher Scientific) and Experion electrophoresis system (Bio-Rad Laboratories). cDNA was synthesized from RNA (400 ng) with the High-Capacity cDNA Reverse Transcription Kit (Applied Biosystems, Foster City, CA, USA). Real-time amplification was done with RT2 SYBR Green qPCR Mastermix (Qiagen) and a ViiA 7 Real-Time PCR System (Thermo Fisher Scientific) as described.^23^ Primers used are shown in Table S3B. Expression was calculated by the ddCt method.

#### Nuclear extract preparation and affinity immunoprecipitation

THP1 cells were cultured to a density of approximately 800,000 cells/mL. The cells were lysed with fractionation buffer (Hepes (pH7.4) 20 mM, KCl 10 mM, MgCl_2_ 2 mM, EDTA 1 mM, EGTA 1 mM) containing protease and phosphatase inhibitors (Pierce #A32959). Nuclei were separated from cytosol, mitochondria and cell membranes by centrifugation, and washed. The nuclei were lysed with a modified RIPA lysis buffer (Tris-HCl pH 7.4 25 mM, NaCl 200 mM, EDTA 1 mM, NonidetP-40 1%, glycerol 5%) containing protease and phosphatase inhibitors.

IP was performed with antibodies recognizing Survivin (ab192675, Abcam, Cambridge, UK), IRF1 (sc74530, Santa Cruz Biotechnology), and SMAD3 (sc101154, Santa Cruz Biotechnology) using the Dynabeads Protein G Immunoprecipitation Kit (10007D, Thermo Fisher Scientific) including cross-linking with bis(sulfosuccinimidyl)suberate (Pierce, Thermo Scientific™ #39266). Control IP was done with non-targeting rabbit IgG (Dako X0902), and mouse IgG (BioLegend 400102).

#### Western blotting

For Western blotting, 30 mg of total nuclear extract and the IP material was separated on NuPage 4–12% Bis–Tris gels (Novex). Proteins were transferred to polyvinylidene difluoride membranes (iBlot, Invitrogen), blocked with a solution of Tris-buffered saline containing Tween-20 and 3% bovine serum albumin, and incubated first with antibodies against IRF1 (H-8, sc-74530), IRF8 (E-9, sc-365042), JUND (D-9, sc-271938), SMAD3 (38-Q, sc-101154), MAX (H-2, sc-8011), and MYC (9E10, sc-40) (all from Santa Cruz Biotechnology; 1:500) and then with peroxidase-conjugated anti-mouse antibodies (NA931, GE Healthcare, Chicago, IL; 1:4000). Bands were visualized with ECL Select Western Blotting Detection Reagent (Amersham) and a ChemiDoc imager and Quantity One software (Bio-Rad Laboratories).

#### Cytokine measurement

Cytokine levels were measured with a sandwich enzyme-linked immune assay as below. Briefly, high-performance 384-well plates (Corning Plasticware, Corning, NY, USA) were coated with capture antibody, blocked, and developed according to the manufacturers’ instructions. Developed plates were read in a SpectraMax340 Microplate reader (Molecular Devices, San Jose, CA, USA) at the dual wavelength of 450/650 nm, and absolute protein levels were calculated after serial dilutions of the recombinant protein provided by the manufacturer. The following reagents were used, for IFNγ (detection limit 3 pg/mL, PelikineM1933, Sanquin, Amsterdam, The Netherlands), IL10 (detection limit 15 pg/mL, DY217B, R&D Systems), IL9 (detection limit 1 pg/mL, DY209, R&D Systems).

#### Glucose uptake assay

CD4^+^ cells were cultured in RPMI medium in anti-CD3 coated plates (0.5 mg/mL) and activated with IFNγ (50 ng/mL) and YM155 (0 and 10 nM) for 24 h. Cells were washed and starved in glucose-free RPMI-medium for 2 h and then supplemented with 2-NBDG (100 μM, Abcam). 2NBDG uptake was registered after 30 min using flow cytometry (Verse, BD) and quantified as the ratio of mean fluorescence intensity to baseline.

### Quantification and statistical analysis

#### RNA-seq analysis

Transcripts were mapped with the UCSC Genome Browser using the annotation set for the hg38 human genome assembly and analyzed with the core Bioconductor packages in R-studio (v.3.6.3). DEGs were identified with DESeq2 (v.1.26.0) with Benjamini-Hochberg adjustment for multiple testing. Volcano plots were prepared with EnhancedVolcano (v.1.4.0). Correlation analysis was done with Hmisc (v.4.5), and the correlation heatmap was built with Corrplot (v.0.85). RNA-seq data were clustered with the Spearman correlation for distance (factoextra, v.1.0.7). WardD2 was used for hierarchical clustering.

#### ChIP-seq analysis

The fastq sequencing files were mapped to the human reference genome (hg38) with the STAR aligner^79^; the alignIntronMax flag was set to 1 for end-to-end mapping. The quality of sequenced material was assessed with the FastQC tool and MultiQC (v.0.9dev0) (Babraham Institute, Cambridge, UK). Peaks were called with MACS2 algorythm for narrow peaks and default parameters. Peaks were filtered for the survivin antibody IP fraction (IP) and unprocessed DNA (Input), which is a generally accepted normalization approach to identify protein-specific enrichment of DNA interaction areas.^80^ A set of peaks with enrichment versus surrounding region and Input (adjusted p < 10^−5^) was identified and quantified separately for each sample. Peaks that overlapped by at least 1 nucleotide in several samples were merged as survivin-ChIP peaks. Peaks in all samples were scored by the number of tags of difference between IP and Input (average of these differences between samples). Peaks were annotated with HOMER software^81^ in standard mode to the closest TSS with no distance restriction. HOMER (findMotifsGenome.pl) and the homer2 engine were used for *de novo* motif discovery and motif scanning. The most common *de novo* motifs were identified separately for each IP sample and examined for detected motifs in the JASPAR database of human TF binding sites.^82^ The Input bed regions were compared with random global controls generated by the service to match the input dataset. For analysis we selected the following binding motifs: JUN.MA0488.1, JUND_2.MA0492.1, IRF1.MA0050.2 and the combined SMAD2_SMAD3_SMAD4.MA0513.1. For each motif, we estimated global control-based p value and fold enrichment. For association with open chromatin, survivin-ChIP peaks within 10 kb from ATAC-seq peaks were detected in CD4^+^ T cells after 2 or 4 h of stimulation.^40^

Genome UCSC annotation hg38 (http://genome.ucsc.edu/) was used to compare the whole-interval set of survivin-ChIP peaks with the set of functional genomic regions. TSSs were defined based on chromStart or chromEnd positions in GENCODE v36. Promoters were defined as regions 5 kb upstream plus 1 kb upstream of the TSS annotated as above. The CTCF-binding sites were accessed according to ENSEMBL regulatory build (v103, 2020 (http://www.ensembl.org/info/docs/funcgen/regulatory_build.html) (177,376 elements). Insulator sites for all aggregated cells were defined according to ENCODE v5, 2020) (https://screen.encodeproject.org/, https://api.wenglab.org/screen_v13/fdownloads/GRCh38-ccREs.CTCF-only.bed file) (56,766 elements). Enhancers were selected with the integrated GeneHancer database (v4.4, https://www.genecards.org/GeneHancer_version_4-4; accessed January 5, 2021. GH score >0.7).

For genomic interval datasets, including survivin-ChIP peaks and REs, the Table Browser for the hg38 human genome assembly (http://genome.ucsc.edu/cgi-bin/hgTables) and Galaxy suite tools (https://usegalaxy.org/) were used for estimating distances between nearest intervals, merging, overlapping, calculating genomic coverage, and other standard procedures. The genome-wide distribution of survivin-ChIP peaks was initially screened with the *cis*-regulatory annotation system (CEAS v0.9.8; accessed November 1, 2020 with Cistrome Galaxy, http://cistrome.org/ap/root). For enrichment analysis, we used the list of all survivin-ChIP peaks and the fraction of them located within 100 kb of the known genes. To estimate pairwise distances and statistical significance of pairwise interval overlaps for survivin-ChIP peaks with genome elements defined above, we used Bedtools suite (https://github.com/arq5x/bedtools2; accessed 01feb2021–15apr 2021). For each comparison, a pairwise, two-tailed Fisher’s exact test was used. Comparison was based on initial survivin-ChIP peak positions as intervals and extended regions with 1-kb, 10-kb and 50-kb flanks.

#### Computational analysis

To identify transcription regulators near survivin-ChIP peaks, we used the ReMap database (http://remap.univ-amu.fr/; accessed November 15, 2020) for colocalization analysis of aggregated cell- and tissue-agnostic human ChIP-seq datasets of 1034 transcriptional regulator. ReMapEnrich R-script (https://github.com/remap-cisreg/ReMapEnrich; accessed November 15, 2020) was used for colocalization enrichment analysis. The hg38 human genome assembly was used for all comparisons. Two-tailed p values were estimated and normalized with the Benjamini-Yekutielli test, using the maximal allowed value of shuffled genomic regions for each dataset (n = 15), kept on the same chromosome (shuffling genomic regions parameter byChrom = TRUE). The default fraction of minimal overlap for input and catalogue intervals was set to 10%. Bed interval files of survivin-ChIP peaks with 0- and 100-kb flanks were prepared. The dataset with 0-kb flanks was compared with the Universe sets of genomic regions, defined as within 1 Mb of the same ChIP-seq peaks. For analysis of the regulatory chromatin paired with DEGs, input bedfiles were selected according to their distance from the genome region containing REs paired to DEGs; bedfiles for individual TFs were downloaded from ReMap2020.

TFs with statistically significant enrichment of overlaps (q value < 0.05, n > 100) were selected. TFs that were enriched with respect to the genomic background were identified within each RE by using ReMap database, as described above. A subset of TFs enriched within the survivin-associated REs was identified by chi-square test (chisq.test, R-studio) and false-discovery rate correction (R-studio). To explore the involvement of these TFs in regulating DEGs, we prepared the presence matrix (1/0 type), excluded regions with 0 overlaps with top TFs, and did a principal component analysis with singular value decomposition imputation. Hierarchical clustering of TFs was done with Canberra or Euclidean distances (prcomp, hclust, R-studio). False discovery rate–adjusted *p* values and the ratio between the survivin-associated and survivin-independent REs per 1 Mb was estimated.

DEGs were compared to all protein-coding human genes (by default) by gene set enrichment analysis (https://www.gsea-msigdb.org/gsea/index.jsp; accessed November 15, 2020). Transcriptional regulators with significant overlap between ChIP-seq and survivin-ChIP peaks were analyzed in comparison to all 1034 transcriptional regulators in ReMap2020. The list of genes corresponding to these regulators was used for functional enrichment analysis in Gene Ontology Biological Processes (GO:BP) using the total list of ReMap transcription regulators as a custom background. Enriched GO:BP categories were grouped together and visualized on the 2D map based on their semantic similarity to show the functional preferences of potential survivin-associated transcriptional regulators. Grouping was carried out by medium term similarity of 0.7 and using ReViGo service (http://revigo.irb.hr/, accessed 01dec2020).

Known genetic associations of the analyzed regulatory regions of DEGs were examined with NHGRI’s collection of GWAS (http://genome.ucsc.edu/). All published GWAS SNPs were included without p value or ancestry filtering. A subset of relevant SNPs was selected by keyword searches for traits of individual autoimmune disorders in Table Browser.^83^

Primary functional chromatin segmentation was accessed by using NIH Roadmap Epigenomic Project data for intact CD4^+^CD25^−^ Th Primary cells (E043 PrimaryHMM; accessed with the Washington University Epigenomic Browser http://epigenomegateway.wustl.edu/browser/roadmap/on March 22, 2022). The default color scheme was applied to chromatin segments of active enhancers, transcribed regions, and repressed and poised loci.

## Data Availability

RNA sequencing data and survivin ChIP-Seq data (raw data and processed files) of CD4^+^T cells have been deposited at GEO and are publicly available as of the date of publication. Accession numbers are listed in the key resources table. This paper does not report original code. Any additional information required to reanalyze the data reported in this paper is available from the lead contact upon request.

## Supplemental information

Document S1. Figures S1–S6 and Tables S1 and S3

Table S2. Complete list of significantly enriched Gene Ontology biological processes (p < 0.001) regulated by TFs that co-localized with survivin ChIP-seq peaks, related to Figure 2 Functional annotation was done in MetaScape. Coordinates for the Gene Ontology biological processes correspond to the x axis and y axis of the semantic map in Figure 2E
