# Supplementary material for: Survivin promotes a glycolytic switch in CD4+ T cells by suppressing the transcription of PFKFB3 in rheumatoid arthritis
Source: iScience. 2022 Nov 7;25(12):105526. doi: 10.1016/j.isci.2022.105526 (PMC9678772; doi:10.1016/j.isci.2022.105526)
Supplement: Document S1. Figures S1–S6 and Tables S1 and S3 [file mmc1.pdf]

iScience, Volume *25*

Supplemental information

Survivin promotes a glycolytic switch in $CD4^+$ T cells by suppressing the transcription of PFKFB3 in rheumatoid arthritis

Malin C. Erlandsson, Karin M.E. Andersson, Nina Y. Oparina, Venkataragavan Chandrasekaran, Tibor Saghy, Anastasios Damdimopoulos, Maria-Jose Garcia-Bonete, Zakaria Einbeigi, Sofia T. Silfverswärd, Marcela Pekna, Gergely Katona, and Maria I. Bokarewa

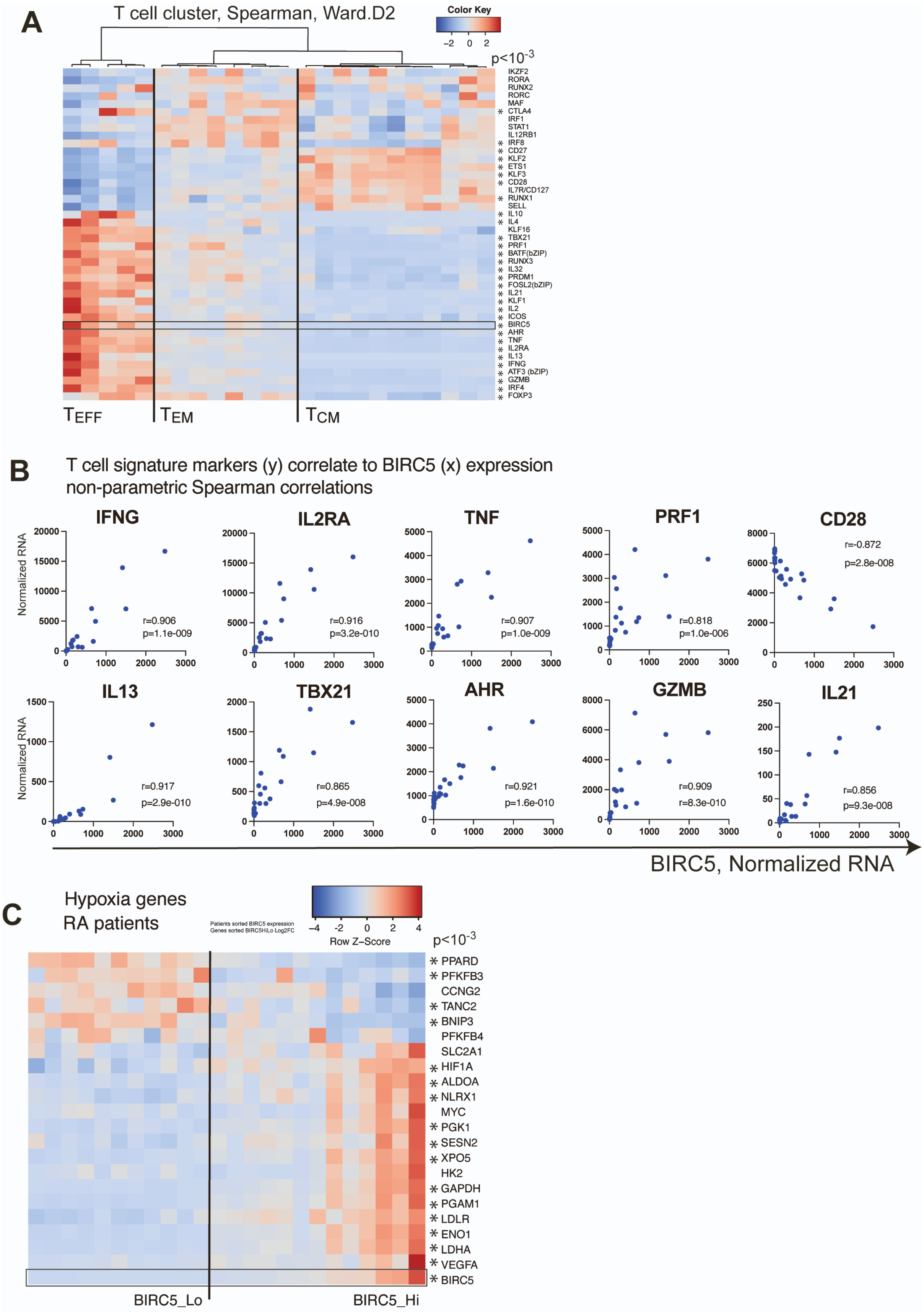

Supplementary Figure S1, related to Figure 1. **(A)** Heatmap of Th1 signature gene expression in CD4+ cells of 24 RA patients by RNA-seq. Samples were clustered unsupervised

by the Spearman-Ward2 method, which resulted in Teff, Tcm and Tem $CD4^+$ cell clusters. DESeq2 was used to compare T_{EFF} and T_{CM} $CD4^+$ cells. Asterisks (*) indicate nominal $p<0.001$. **(B)** Spearman correlation analysis of *BIRC5* normalized RNA-seq values between *BIRC5* expression and Th1 signature genes. *IFNG*, interferon γ; *IL2RA*, interleukin 2 receptor α; *TNF*, tumor necrosis factor; *PRF1*, perforin 1; *TBX21*, T-box transcription factor 21; *AHR*, aryl hydrocarbon receptor; *GZMB*, granzyme B. (**C)** Heatmap of normalized RNA-seq values of the hypoxia sensitive genes in $CD4^+$ cells of 24 RA patients. Samples are ranged after *BIRC5* mRNA levels. $BIRC5^{hi}$ and $BIRC5^{lo}$ (median split) $CD4^+$ cells were compared with DESeq2. Asterisks (*) indicate nominal p<0.001.

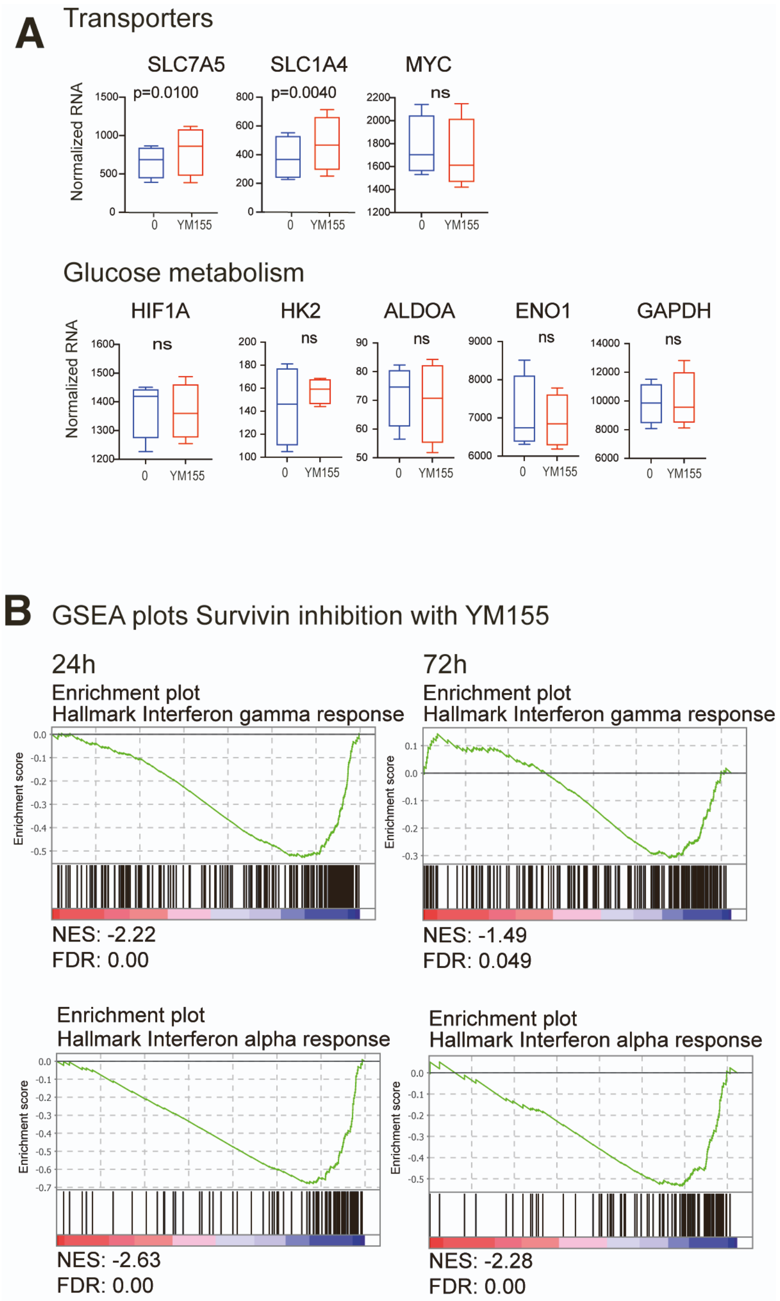

Supplementary Figure S2, related to Figure 3. (A) Box plots of expression by RNA-seq values of genes to hypoxia sensors, glucose metabolism, sugar sensors, and transporters in $CD4^+$ cells treated with YM155 (0 and 10nM) for 24h. (**B)** Barcode plots of the IFNα and IFNγ signaling pathway for DEGs in YM155-treated $CD4^+$ cells, by GSEA.

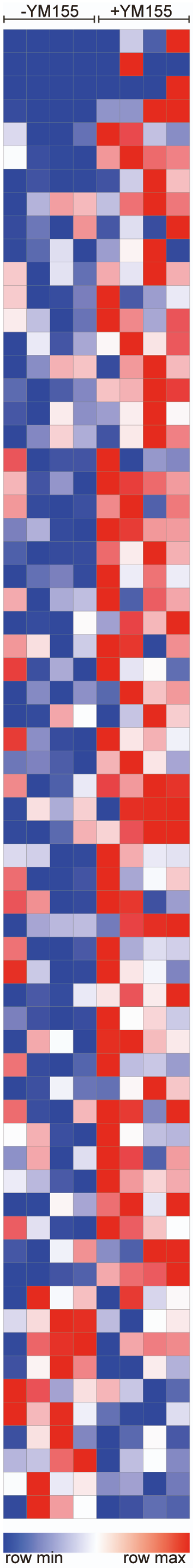

	log2FC	pvalue	padj	baseMean
SEZ6L2	19.49	1.2E-11	7.1E-08	0.81
CCDC73	19.48	4.0E-06	2.7E-03	1.18
RPA4	15.42	2.9E-04	3.8E-02	0.89
DTL	3.40	2.9E-03	2.0E-01	3.73
GJC3	2.11	4.2E-03	2.8E-01	9.56
FLT3LG	0.78	1.3E-04	2.1E-02	96.69
QPCT	0.78	2.3E-03	1.6E-01	58.48
PERP	0.57	4.6E-03	3.1E-01	93.27
CDK5R1	0.57	2.2E-11	1.1E-07	817.05
RGS16	0.56	4.1E-04	4.3E-02	143.96
SPSB1	0.55	3.0E-04	3.8E-02	408.29
SIAH3	0.53	5.0E-04	4.5E-02	213.96
TGFA	0.48	1.0E-04	1.7E-02	260.79
DIPK2A	0.47	2.0E-03	1.4E-01	141.65
FOXP4	0.45	3.9E-03	2.7E-01	287.71
PMEPA1	0.45	3.0E-08	5.3E-05	1082.49
MYO1C	0.44	3.3E-04	4.0E-02	487.43
FAM102B	0.38	8.9E-06	4.9E-03	571.60
CTSL	0.37	1.7E-07	2.3E-04	2771.17
PGM2L1	0.37	6.4E-04	5.5E-02	546.41
NKD1	0.37	2.4E-03	1.7E-01	1047.79
SMURF2	0.37	6.7E-12	5.1E-08	1690.91
CAMSAP2	0.36	1.2E-03	9.2E-02	371.48
ESCO1	0.35	1.2E-04	2.0E-02	586.52
SLC25A53	0.35	1.5E-04	2.3E-02	473.97
MIDN	0.35	2.4E-04	3.4E-02	877.02
HVCN1	0.34	2.4E-04	3.3E-02	434.91
FBLN7	0.33	8.3E-04	6.7E-02	758.81
SKI	0.32	2.6E-05	9.4E-03	4298.63
SLC1A4	0.31	4.0E-03	2.7E-01	420.30
PFKFB3	0.30	7.1E-06	4.1E-03	3514.83
SKIL	0.28	1.1E-03	8.6E-02	1504.85
SAMD1	0.27	5.5E-04	4.9E-02	1183.18
IL21R	0.27	2.0E-03	1.4E-01	1260.02
ZFP90	0.27	2.2E-04	3.2E-02	883.26
BIRC2	0.27	5.2E-07	5.6E-04	2415.67
BPGM	0.27	1.3E-03	9.8E-02	684.36
NET1	0.26	1.5E-03	1.1E-01	629.90
SMAD7	0.24	2.1E-03	1.5E-01	1698.88
SLC20A2	0.23	1.0E-03	7.8E-02	927.57
GRAP	0.23	4.7E-03	3.1E-01	2269.68
ELK3	0.23	6.1E-05	1.2E-02	1754.60
KTN1	0.22	3.1E-04	3.9E-02	3083.67
EMB	0.21	7.8E-04	6.4E-02	3468.08
OCIAD2	0.20	3.5E-03	2.4E-01	2137.80
LDLRAD4	0.19	6.2E-04	5.4E-02	1846.27
ZFP36L1	0.18	1.5E-03	1.1E-01	4813.16
STK17A	0.18	4.3E-03	2.9E-01	2605.05
RBMS1	0.18	4.4E-03	3.0E-01	1638.55
PDCD4	0.17	2.1E-04	3.1E-02	7200.94
HNRNPA3	0.17	4.0E-03	2.7E-01	3206.42
TES	0.16	2.8E-03	1.9E-01	2783.57
CERK	0.15	1.8E-03	1.3E-01	7531.03
HNRNPA2B1	0.14	1.4E-03	1.0E-01	11237.13
GNLY	-0.21	1.5E-03	1.1E-01	3674.03
PYHIN1	-0.26	2.1E-03	1.5E-01	822.14
PRF1	-0.29	7.4E-05	1.3E-02	1319.28
ARID5B	-0.33	6.4E-05	1.2E-02	875.21
TMEM25	-0.34	2.0E-03	1.4E-01	336.06
NLRX1	-0.39	4.1E-03	2.8E-01	192.97
TTYH2	-0.41	1.0E-03	7.7E-02	339.32
SESN2	-0.48	3.3E-03	2.3E-01	164.43
UBE2T	-0.85	3.2E-04	4.0E-02	57.68
CXCL8	-2.08	2.5E-03	1.8E-01	14.75

Supplementary Figure S3, related to Figure 4. Heatmap of 77 protein-coding DEGs (RNA-seq normalized values, base mean >0.5, DESeq2 nominal *p*-value <0.005) in CD4^{+} cells treated with YM155 for 24 h and stimulated with IFNγ for the last 2 h (n=4 healthy controls).

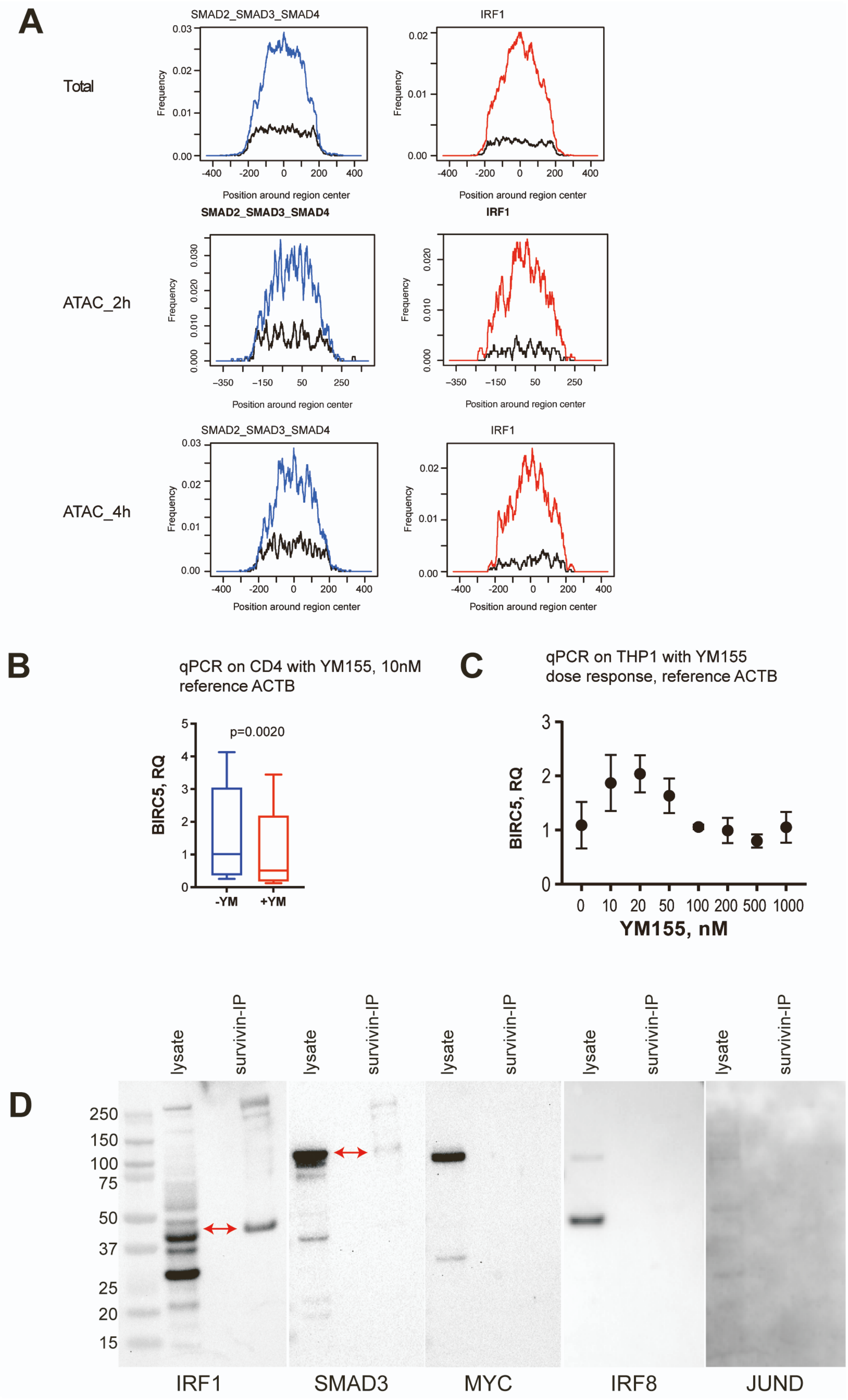

Supplementary Figure S4, related to Figure 4. **(A)** Histograms profiles of frequency distribution for IRF1 and SMAD2/SMAD3/SMAD4 binding motifs in the total set of survivin-

ChIP peaks across the genome and in ATAC-seq annotated open chromatin regions in CD4^{+} cells activated for 2 h and 4 h. Black line indicates matched random controls. (**B)** Expression of *BIRC5* in primary CD4+ and THP1 cells, by RT-PCR. (**C)** *BIRC5* and *PFKFB3* mRNA levels in THP1 cells treated with increasing concentrations of YM155 for 48h, by RT-PCR. (**D)** Western blots of THP1 cell lysate before and after affinity immunoprecipitation with survivin, stained for IRF1, SMAD3, MYC, IRF8 and JUND.

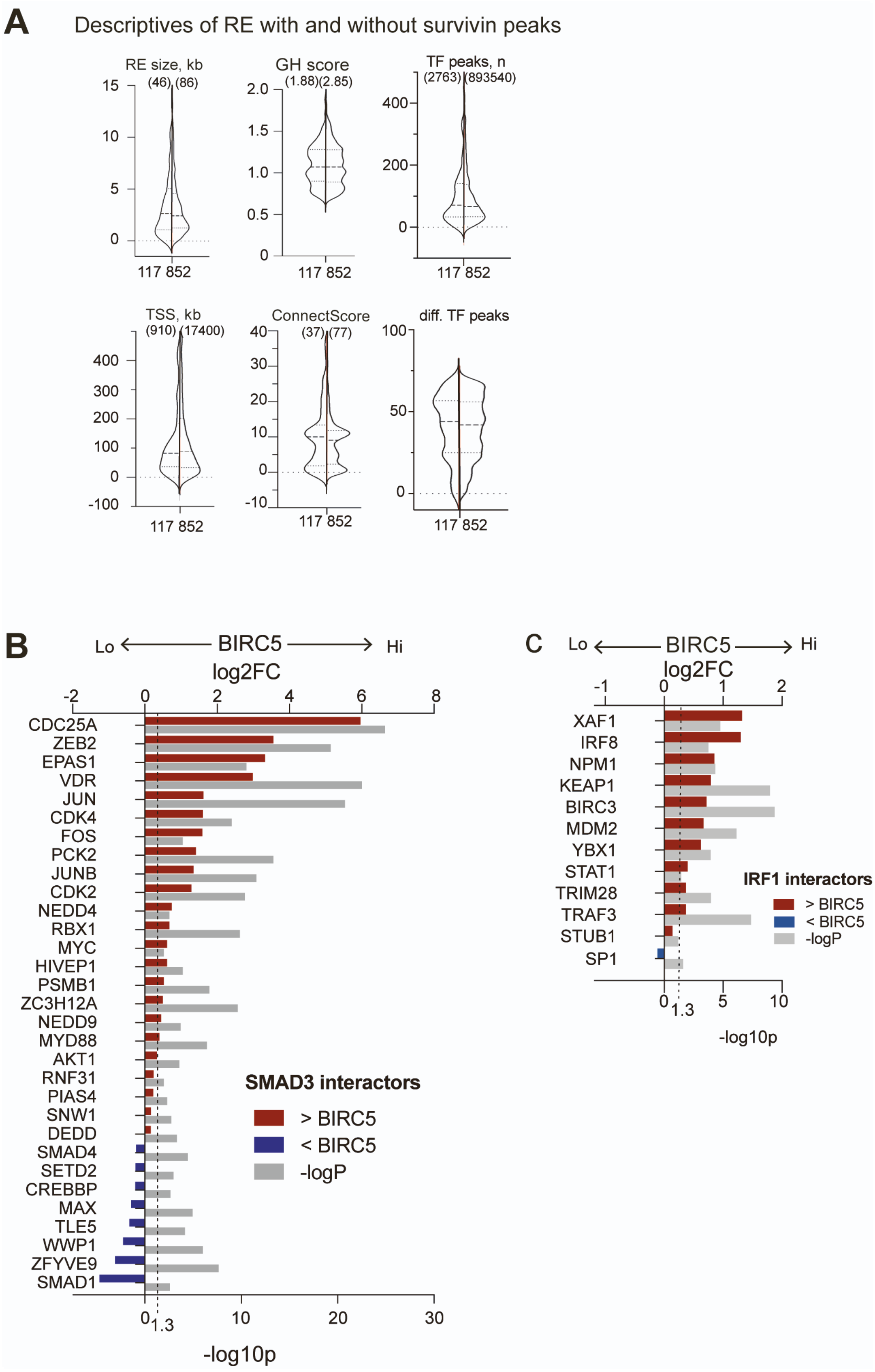

Supplementary Figure S5, related to Figure 5. (A) Violin distribution plots of specific characteristics of REs paired to survivin-sensitive DEGs with the GeneHancer database. REs

containing survivin-ChIP peaks (*n* = 117) and the remaining RE (*n* = 852) were compared by length, distance to TSS, GeneHancer score, GeneConnect score, and number of TFs per RE. *P*-values were determined by unpaired *t* test. (**B, C)** Forest plot of enrichment and *p*-values for IRF1 **(B)** and SMAD3 **(C)** interactors (BioGrid database, ≥2 physical evidence) in *BIRC5*^{hi} and *BIRC5*^{lo} $CD4^{+}$ cells in 24 RA patients. RNA-seq data were analyzed with DESeq2. Nominal *p*-values are indicated.

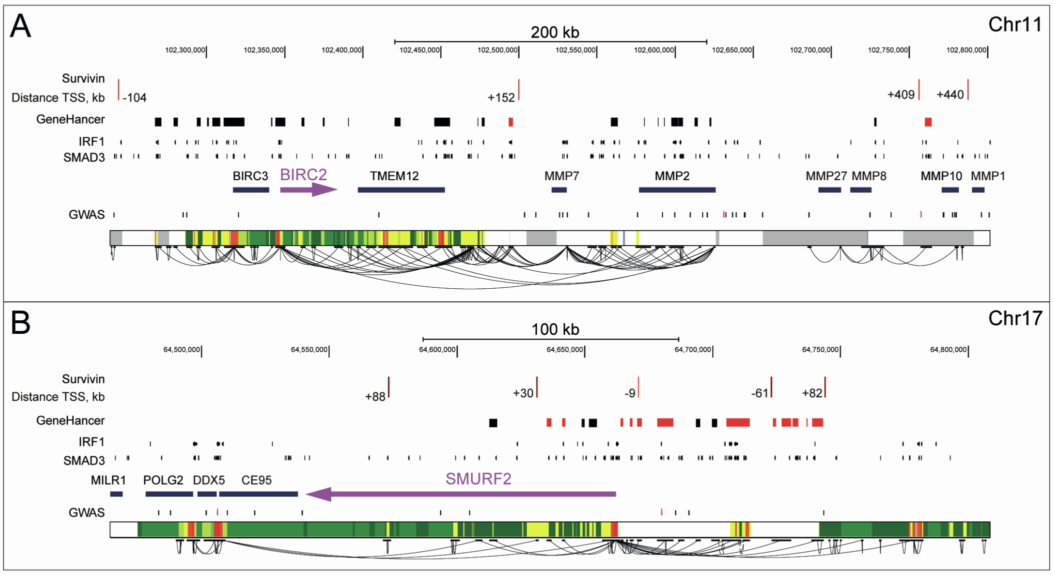

Supplementary Figure 6S, related to Figure 6. Genomic maps of *BRIC2* **(A)** and *SMURF2* ***(B)*** gene loci**.** Magenta indicates position of the canonical gene transcript; arrow indicates transcription orientation. Red dashes at the top of each locus indicate the positions of survivin-ChIP peaks. Distances to TSSs are shown. Boxes indicate REs paired to the gene, as determined with GeneHancer. Solid curved lines indicate integrated annotation of RE connections identified with GeneHancer. Red boxes indicate REs <10 kb from the survivin peak. Vertical lines indicate positions of ChIP-seq peaks for IRF1 and SMAD3 determined by ReMap2020. Vertical lines indicate positions of GWAS SNPs associated with metabolic and autoimmune triads according to NHGRI GWAS catalog. Functional chromatin segmentation for intact $CD4^{+}$cells (RoadMap ChromHMM. E043:$CD4^{+}CD25^{-}$) is shown at the bottom of each map. Green blocks indicate actively transcribed areas; yellow blocks indicate enhancers; red blocks indicate active promoters; white blocks indicate areas of repressed/poised chromatin.

Supplementary Table S1, related to Figure 1. Clinical characteristics of RA patients

Variable	Flow cytometry, PBMCs ($n = 22$)	RNA-seq, $CD4^+$ T cells ($n = 24$)
Mean age, years (range)	62.2 (37–73)	55 (46.5–66)
Female, n (%)	16 (72.7)	24 (100)
Mean disease duration, years (range)	19.5 (9–47)	5.5 (1.75–11.25)
RF/ACPA positive, n (%)	17 (77.3)	16 (66.7)
Treatment		
Methotrexate, n (%)	20 (90.9)	16 (66.7)
Anti-TNF, n %	20 (90.9)	5 (20.8)
Other biologics, n (%)	2 (9.1)	4 (16.7)

TNF, tumor necrosis factor. Arthritis specific antibodies, rheumatoid factor (RF) and antibodies to cyclic citrullinated peptides (ACPA)

Suppplementary Table S2, Related to Figure 2. Complete list of significantly enriched Gene Ontology biological processes ($p < 0.001$) regulated by TFs that colocalized with survivin ChIP-seq peaks
Functional annotation was done in MetaScape. Coordinates for the Gene Ontology biological processes correspond to the *x*-axis and *y*-axis of the semantic map in Fig. 2E.

The table is available in Excel format

Supplementary Table S3A, related to Figure 6. Primers used for amplification from chromatin immunoprecipitated material.

Primer code	Sequence	Position	Tm primer	Amplicon size	Amplicon sequence
F1	GCCCTTCCCATCACAGTGAA	chr10|6130121|6130140	59.96	176 bp	GCCCTTCCCATCACAGTGAATGTTGC CAGTTGGGGATGACTGGGCAGCCAT GGGGAAGGTGAGCAGAGGGCAGAG GAGTCCAAGCCCCTGAGGTGGAGGT GGGAGCAGGGAGAGCTGCAGGCTCA GCTGCTGGGGCAGTAGGAGGAGAGC CAGGACCTGGAGGAGTGTGTGTGTGT
R1	ACACACACACACTCCTCCAG	chr10|6130277|6130296	59.53		
F2	AAACCCTCTTCCATGCCAGA	chr10|6404335|6404354	58.92	197bp	AAACCCTCTTCCATGCCAGACTCTGA CAATGGATTCATTCTTTCTTTCGTTCT TTCTTTTCTTTCTTTCCTTCTTTCTTTC TTTTTTCTCTTTCTTTCTCTCTTTCTTTC TCTTTCCTTTCTTCCTTCCTTTTTTCCT TTTTTCTTTCTTTCTCTCTTTCCTTTCT TTCTCTGTCTCTCCTTCCTCCCTCCTTC CTT
R2	AAGGAAGGAGGGAGGAAG GA	chr10|6404512|6404531	59.20		
F3	TGCAGAATTCTACCCTTTCC TTG	chr10|6404132|6404154	58.66	172bp	TGCAGAATTCTACCCTTTCCTTGCCA AGATTAGATAAGGGCAAAAATCCTT TAATGGCATTAAAGAGAAAGAGAAAA GAAAAAACTAGAGAGAGAGAGAG AGAGAAAGGGAGAGAAGGGGGGGG GAGAGAGAGAGAGAGAGAGAGAG AGAGAGAGATTGCATGGCCAGAGGT
R3	ACCTCTGGCCATGCAATCT	chr10|6404285|6404303	58.99		
F ctrl	GGTGTGCAGGATAACGGAG T	chr10|6201978|6201997	60.0	135bp	GGTGTGCAGGATAACGGAGTGAATTT TAGGAGTCTGCCTAATAAGGGGCCGCC CCCAATAGTTTTCCCTCGACTTAGGTA ATGAATTGCAGATCGAAATGGGTCTAG TTTTATTCATTCTTCTACTCGGGGCGAT
R ctrl	ATCGCCCCGAGTAGAAGAAT	chr10|6202093|6202112	60.1		

Supplementary Table S3B, related to Figure 4. Primers for quantitative PCR analysis

Target	Forward	Reverse
SLC2A1	ACTGGAGTCATCAATGCCCC	AGAAGGAGCCAATCATGCCC
PFKFB3	CCTACAACTTCTTCCGCCCC	CCGCAATTTGTCCCCCTTCT
BIRC5	GACCACGCCATCTCTACATTC	TGCTTTTTATGTTCCTCTATGGG
